# *De Novo* Transcriptome Analysis of the Common New Zealand Stick Insect *Clitarchus hookeri* (Phasmatodea) Reveals Genes Involved in Olfaction, Digestion and Sexual Reproduction

**DOI:** 10.1371/journal.pone.0157783

**Published:** 2016-06-23

**Authors:** Chen Wu, Ross N. Crowhurst, Alice B. Dennis, Victoria G. Twort, Shanlin Liu, Richard D. Newcomb, Howard A. Ross, Thomas R. Buckley

**Affiliations:** 1 Landcare Research, Auckland, New Zealand; 2 School of Biological Sciences, The University of Auckland, Auckland, New Zealand; 3 New Zealand Institute for Plant & Food Research Ltd, Auckland, New Zealand; 4 Institute of Integrative Biology, ETH Zürich, Zürich, Switzerland; 5 EAWAG, Swiss Federal Institute of Aquatic Science and Technology, Dübendorf, Switzerland; 6 China National GeneBank, BGI-Shenzhen, Shen Zhen, China; USDA-ARS, UNITED STATES

## Abstract

Phasmatodea, more commonly known as stick insects, have been poorly studied at the molecular level for several key traits, such as components of the sensory system and regulators of reproduction and development, impeding a deeper understanding of their functional biology. Here, we employ *de novo* transcriptome analysis to identify genes with primary functions related to female odour reception, digestion, and male sexual traits in the New Zealand common stick insect *Clitarchus hookeri* (White). The female olfactory gene repertoire revealed ten odorant binding proteins with three recently duplicated, 12 chemosensory proteins, 16 odorant receptors, and 17 ionotropic receptors. The majority of these olfactory genes were over-expressed in female antennae and have the inferred function of odorant reception. Others that were predominantly expressed in male terminalia (n = 3) and female midgut (n = 1) suggest they have a role in sexual reproduction and digestion, respectively. Over-represented transcripts in the midgut were enriched with digestive enzyme gene families. *Clitarchus hookeri* is likely to harbour nine members of an endogenous cellulase family (glycoside hydrolase family 9), two of which appear to be specific to the *C. hookeri* lineage. All of these cellulase sequences fall into four main phasmid clades and show gene duplication events occurred early in the diversification of Phasmatodea. In addition, *C. hookeri* genome is likely to express *γ*-*proteobacteria pectinase* transcripts that have recently been shown to be the result of horizontal transfer. We also predicted 711 male terminalia-enriched transcripts that are candidate accessory gland proteins, 28 of which were annotated to have molecular functions of peptidase activity and peptidase inhibitor activity, two groups being widely reported to regulate female reproduction through proteolytic cascades. Our study has yielded new insights into the genetic basis of odour detection, nutrient digestion, and male sexual traits in stick insects. The *C. hookeri* reference transcriptome, together with identified gene families, provides a comprehensive resource for studying the evolution of sensory perception, digestive systems, and reproductive success in phasmids.

## Introduction

Stick insects (Phasmatodea) are a diverse group of insects with over 3000 described species. They occur throughout much of the world, with the greatest species diversity occurring in tropical and subtropical regions [1]. They are experts in camouflage, as both their morphology and movements resemble twigs and foliage. Phasmids across the globe have been the subject of studies on a variety of traits, including the patterns of geographic variation and evolution, sensory control of movement, variation of reproductive modes, evolution of body form, and intestinal digestion [2–8]. Most recently the genus *Timema*, endemic to North America, has become a successful model for addressing evolutionary questions, especially surrounding the genomic basis of speciation [9, 10]. However, compared with insects from other orders, the molecular basis of phasmid biological function has been poorly studied, impeding our understanding of interesting phasmid traits.

Next generation sequencing (NGS) methods such as RNA-Seq now provide the opportunity to greatly enrich molecular resources for non-model species. The relative ease with which RNA-Seq can be utilized in both *de novo* assembly and read mapping to assembled transcripts facilitates the exploration of gene diversity and expression in non-model organisms. Such technology has already been applied to stick insects in a few instances [5, 8, 11–16] with only four of them focusing on the study of phasmid functional traits. Midgut transcriptomes were sequenced to examine plant cell wall degrading enzymes (PCWDEs) in *Peruphasma schultei*, *Sipyloidea sipylus*, *Aretaon asperrimus*, *Extatosoma tiaratum*, *Medauroidea extradentata* and *Ramulus artemis* [5]. The presence of cellulase (GH9), cellobiase (GH1), pectinase (GH28) and *β*-1,3-glucanase (GH16) transcripts in the stick insect anterior midgut demonstrated that, unlike some insects that rely on bacterial symbionts to digest some components of PCWs, these stick insects have the ability to independently digest cellulose into glucose and also other components such as pectin and chitin substrates to obtain carbon [5, 17, 18]. More recently, exploration of RNA-Seq from *Phyllium siccifolium* antennae identified olfactory receptors (ORs), ionotropic receptors (IRs) and gustatory receptors (GRs), revealing a fully developed olfactory receptor-based system in phasmids [14]. Also, to investigate expression of the genes associated with cold temperature adaptation in the alpine New Zealand stick insect species, RNA-Seq was used to sequence heads and prothoraxes of both alpine and lowland species including those belonging to the genera *Micrarchus*, *Tectarchus*, *Niveaphasma*, *Asteliaphasma*, *Argosarchus*, *Spinotectarchus*, *Acanthoxyla* and *Clitarchus* [12, 13, 15]. Despite these studies, transcriptomic data from stick insects has still not been collected comprehensively across tissues, sexes, and species.

The New Zealand stick insect fauna includes 24 species from 10 genera, and are thought to have originated from New Caledonia approximately 24–45 million years ago [19, 20]. They now occupy habitats from the cold alpine zone to dry coastal scrub across the country [15, 21]. *Clitarchus hookeri* is one of the most common New Zealand stick insect species, and is widely distributed throughout the North Island and across large areas of the South Island [4, 22]. It is an ecological generalist, found in both low altitude forest and scrub habitat [22, 23]. Compared with other New Zealand species, *C. hookeri* has a relatively large body size, with adult females ranging in size from 80 to 106 mm while males are slimmer and shorter (approximately 70 mm) [24]. Their bodies are narrow and mostly cylindrical with smooth or slightly wrinkled exoskeletons, and are often bright green or brown [24]. The main native host plants of *C. hookeri* are *Leptospermum scoparium* (Manuka) and *Kunzea sp*. (Kanuka), but they can also be found on other plants, from the families of *Myrtaceae*, *Polygonaceae*, *Laxmanniaceae* and *Rubiaceae* [4, 22], as well as the introduced species of *Rubus*.

The most genetically varied populations of *C. hookeri* occur in the northern half of the North Island, and this genetic variation decreases dramatically from north to south [4, 22]. It has been suggested that during the Last Glacial Maximum, this species was restricted to several refugia located on the northern half of North Island, followed by subsequent population expansion across the country [22]. Molecular resources from *C. hookeri* are lacking, restricted to a few neutral gene markers that were sequenced and used for phylogenetic construction [4, 19, 20, 22, 25] and a head and prothorax transcriptome that was constructed for the study of cold temperature adaptation [15].

In order to examine digestive enzymes, olfaction-associated proteins and accessory gland proteins in a New Zealand stick insect species, we carried out an RNA-Seq experiment by sequencing the transcriptomes of *C. hookeri* female antennae, midgut and male terminalia. To construct a more complete reference transcriptome for the species for future use, we also integrated the *C. hookeri* composite leg, head and prothorax transcriptomes that have been constructed for the use in other studies.

## Materials and Methods

### Sample information

The *C. hookeri* transcriptome presented in this paper was constructed from five different types of tissue derived from nine adults collected in February 2013. These insects were all from the same colony inhabiting on Manuka/Kanuka trees in Totara Park, Auckland, New Zealand (37°0.111 S, 174°55.039 E) (Table 1). This collection site was on Auckland Council administered land and we collected samples under permits issued by the Auckland Council. We use the term ‘tissue’ to describe all of these samples in the following text for simplicity; although some samples are more accurately described as ‘organs’ or ‘regions of the body’. Insects were directly snap-frozen and stored at −80°C after collection.

**Table 1:** Sample summary.

Tissue type	Sample code	Sex	Assembly available
**Antenna**	CLI602	Female	Current study
**Midgut**	CLI602	Female	Current study
**Terminalia**	CLI601	Male	Current study
**Head and prothorax**	CLI303-305,CLI308-310	Female	Yes [15]
**Leg**	CLI524	Female	Yes (For 1KITE project)

### RNA isolation and deep sequencing

Tissue for RNA extraction was removed using sterilised scalpel blades, with the midgut dissection being carried out in 100% ethanol followed by removal of the gut contents. The RNA extraction and sequencing from head and prothorax is described elsewhere [15]. The leg transcriptome was previously sequenced for the 1KITE project on the ‘evolution of insects’ (NCBI accession number: PRJNA286351); however, the RNA extraction and sequencing have not been described elsewhere. We therefore describe them below.

#### Antennae, midgut and terminalia

RNA extraction was conducted separately on the antenna, midgut and terminalia tissues using the same method. Total RNA was extracted using TRIzol RNA extraction regent (Life Technologies), following grinding in liquid nitrogen, according to the manufacturer’s protocol. Subsequent RNA clean-up was performed using the RNeasy Mini Kit (Qiagen). Quality and concentration of RNA extractions were assessed using a NanoDrop ND-1000 spectrophotometer (NanoDrop Technologies, Wilmington, DE, USA) and an Agilent 2100 Bioanalyzer (Agilent Technologies, Palo Alto, CA, USA). cDNA libraries were constructed using the Illumina TruSeq RNA Sample Preparation Kit v2 (Illumina San Diego, CA, USA) according to the manufacturer’s protocol. Individual libraries had randomly assigned barcodes ligated to the cDNA fragments and were amplified with 12 cycles of PCR. Library quality was assessed using an Agilent 2100 Bioanalyzer. Extractions were pooled together for sequencing on the Illumina HiSeq2000 platform at New Zealand Genomics Limited (http://www.nzgenomics.co.nz/) to generate 100 bp paired end (PE) reads. All reads have been deposited in the Sequence Read Archive (SRA) at NCBI (accession numbers: SRR3080264, SRR3080247, SRR3080266).

#### Leg

Forelegs were preserved in RNAlater and sent to BGI Shenzhen for RNA extraction, library preparation and sequencing. mRNA was isolated using the Dynabeads mRNA Purification Kit (Invitrogen, Grand Island, NY, USA) and subsequently sheared using RNA fragmentation reagent (Ambion, Austin, Texas, US) at 72°C. These cleaved RNA fragments were transcribed into first-strand cDNA using SuperScript^™^II Reverse Transcriptase (Invitrogen, Grand Island, NY, USA) and random N6 primer (IDT). The second-strand cDNA was then synthesized using RNase H (Invitrogen, Grand Island, NY, USA) and DNA polymerase I (New England BioLabs, Ipswich, MA, USA). The double-stranded cDNA then underwent end-repair, a single ‘A’ base addition, adapter ligation, and size selection on agarose gels (250 ± 20 bp). The product was then indexed and PCR amplified to finalize library preparation for the paired-end cDNA. Verification of the cDNA fragment size and concentration was accomplished using an Agilent 2100 Bioanalyzer and an ABI StepOnePlus Real-Time PCR machine. The cDNA library was subsequently sequenced on an Illumina HiSeq2000 with 150 bp PE reads, generating approximately 2.5 Gb raw data.

### *De novo* transcriptome assembly and assessment

The *C. hookeri* transcriptome assembly was produced by combining three *de novo* assemblies: the pooled antennae, midgut and terminalia assembly, and the two previously assemblied leg, and head and prothorax assemblies. The head and prothorax assembly is described elsewhere [15].

#### Antennae, midgut and terminalia

Illumina sequencing reads were pooled together and quality assessed with FastQC (http://www.bioinformatics.babraham.ac.uk/projects/fastqc/). To reduce the effects from inherent sequencing biases resulting from library preparation [26, 27], raw reads were trimmed by removing eight nucleotides (nt) from the 5’ end using PRINSEQ (v.0.20.3) [28]. To maximise quality, reads were then processed to remove ambiguities using PRINSEQ, trim adapters and low quality regions (Phred < 30) using cutadapt (v1.2.1) [29]. Remaining reads that were less than 50 bp in length, and those no longer paired were removed using PRINSEQ.

To optimise the completeness of the transcriptome and the number of full-length transcripts generated from the pooled three libraries, the *de novo* assembly was obtained by combining assembled contigs derived from Trinity (v20140413) [30], SOAPdenovo-trans (v1.03) [31], and Velvet/Oases (v0.2.08) [32]. The Kmer sizes 21, 25, 29, and sizes from 35 to 85 with stepwise increase of ten were used in SOAPdenovo-trans and Velvet/Oases, and scaffold gaps of each assembly were filled by GapCloser (http://sourceforge.net/projects/soapdenovo2/files/GapCloser/). Each final transcript was given an ID consisting of species, assembly version, assembler, Kmer size if applied, and an unique seven digit ID, eg. Chv1VELVK21_0000001.

#### Leg

Raw data were preprocessed by removing reads of poor quality including: 1) reads with adapter; 2) reads with a total number of >10 Ns; 3) reads with >50 base pairs of low quality (Phred quality score = 2, ASCII 66 “B”, Illumina 1.5+ Phred+64). Transcripts were assembled using SOAPdenovo-trans (v1.01) with a Kmer of 31. The gapped sequences were filled using GapCloser. Each contig ID was replaced by the code ChvKITESOAP with a 7 digit ID, which vKITE stands for the assembly version produced for the project of ‘evolution in insects’ (GenBank bioproject accession: PRJNA286351) conducted by 1K Insect Transcriptome Evolution (http://www.1kite.org/).

#### Final assembly and assessment

The final transcriptome of coding transcripts was created by combining a total of 19 assemblies described above which were then run through the EvidentialGene tr2aacds pipeline (http://arthropods.eugenes.org/EvidentialGene/). This pipeline employs CD-HIT-EST (v3.1.1) [33] to cluster transcripts with identity of 90% or greater and then select the ‘best’ contig based on the open reading frame (ORF) and UTRs present from each cluster as the final coding transcript. The resulting transcript set was then quality evaluated with Core Eukaryotic Genes Mapping Approach (CEGMA: v2.4) [34] software, which detects a core protein set of 248 highly conserved proteins that are found in a wide range of eukaryotes. The cleaned reads were also mapped back to both the raw and final transcriptomes using Bowtie2 (v2.1.0) [35] to produce a coverage profile of the assemblies from short reads. To estimate levels of xenobiotic sequence contamination, transcripts were assigned to different taxonomic categories (eg. arthropod, fungi, bacteria, and virus) with Assemblage (https://github.com/sujaikumar/assemblage).

### Gene functional annotation

*De novo* transcripts were annotated using Blastx (v2.2.28) [36] (e-value < 1e^-5^) to search against the GenBank non-redundant (*nr*) protein database (downloaded in April 2015). Transcripts lacking Blast hits were searched for conserved protein domains with InterProScan [37] within Blast2GO (v2.8) [38]. Gene Ontology (GO) terms were assigned using Blast2GO.

### Identification of chemosensory proteins

As many chemosensory genes are often duplicated in a lineage-specific manner and are divergent from previously annotated chemosensory genes at both the nucleotide and amino acid levels, particularly in understudied lineages (eg. phasmids), we employed additional identification steps to optimise the completeness of these proteins in *C. hookeri*. First, the predicted *C. hookeri* initial set of chemosensory transcripts were generated using proteins from closely related insect lineages, if available, to search the homologues against the *C. hookeri* transcriptome assembly (tBlastn, evalue < 10^-5^). The searching set included 29 *Zootermopsis nevadensis* OBPs [39], three *Eurycantha calcarata* CSPs [40], all the *P. siccifolium* ORs, IRs and GRs [14], and two *Epiphyas postvittana* SNMPs [41]. Second, the resulting *C. hookeri* homologous transcripts were translated and used to additionally search the homologues against the transcriptome assembly with the same Blast search. Third, all the predicted *C. hookeri* chemosensory transcripts were searched against the GenBank *nr* protein database (Blastx; e-value < 10) to confirm their protein identities. Putative OBPs and CSPs were additionally confirmed based on the presence of conserved cysteines. Finally, the identified transcripts were aligned to a *C. hookeri* genome assembly (Wu et al. in prep for publication) to rule out mis-assembled sequences.

### Transcript abundance and differential expression analysis

Transcript quantification for each tissue, based on the combined transcriptome, was carried out with RSEM (v1.2.11) [42]. Transcript abundances were measured by mapping short reads to the assembly using Bowtie2 and calculating a maximum likelihood abundance with a credibility interval for counts of genes or isoforms to determine levels of differential expression. Estimates of transcript abundance were produced in the form of two measures, the expected counts (ECS) and transcripts per million (TPM). The ECS is an estimate of the number of fragments that can be derived from an isoform or gene, which is calculated with the maximum likelihood expression levels using an Expectation-Maximisation (EM) algorithm. In this study, transcripts were considered present in the library if ECS was larger than one. TPM estimates the fraction of transcripts within the sample that are represented by the given isoform or gene [43]. A data matrix containing ECS from all libraries was imported into EBSeq (v1.10.0) [44] to find significantly enriched transcripts in one tissue library relative to the others. EBSeq is an R package utilizing an empirical Bayesian approach to model the features observed in RNA-Seq data. It was used as it (i) allows multi-class testing among RNA-Seq datasets and can generate all possible expression patterns given by the conditions rather than only calculating pair-wise comparisons, (ii) estimates cross-condition variance as the transcript variance by pooling similar genes together for the comparison among samples without replicates, and (iii) has been shown to have a relatively low false discovery rate and calculate expression of isoforms more accurately compared with other algorithms that have been used to detect differentially expressed genes [45]. Here, we considered a gene to have tissue-biased expression if it was detected with posterior probability of being differentially expressed (PPDE) greater than 0.95 (this is equivalent to false discovery rate < 0.05). The gene was also considered as significantly over-represented in a tissue library if it achieved a expression fold change of at least one when compared with every other tissue libraries. GO enrichments for each gene set with tissue-biased expression were calculated using Fisher’s Exact Test with multiple testing correction of false discovery rate (Benjamini and Hochberg) less than 0.05 on Blast2GO.

### Phylogenetic analysis

Protein alignments of OBP and olfactory receptor co-receptor (ORCO) were generated using MUSCLE [46] with the default options in Geneious (v7.1.7) [47] and curated using Gblocks (v0.91b) [48]. Rooted phylogenetic trees were constructed using PhyML (v2.0) [49] under the best fit models (OBP: VT+I+G; ORCO: LG+G+F) selected by Prottest (v3.4) [50]. A non-parametric bootstrap analysis was conducted to generate support for branching topology (n = 500 bootstrap pseudoreplicates).

The GH9 cellulase gene phylogeny was constructed using protein coding nucleotide sequences. The GH9 sequences were aligned using MUSCLE translation alignment method with default gap penalty parameters in Geneious. Rooted phylogenetic trees were constructed using PhyML under the best fitting substitution model (TIM2+I+G) selected by JModelTest2 (v2.1.6) [51] with 500 bootstrap pseudoreplicates.

### Tests for branch selection in ORCO

Tests for branch selection were implemented in the CODEML program from PAML (v4.8) [52] using a branch-site model available for an arbitrary number of ratios (model = 2, Nsites = 2). The null and alternative hypothesises for this test were inferred by giving an initial *ω* value of one and an option of estimate respectively. The inference of positive selection on the given phylogenetic branch was conducted using likelihood ration tests (LRTs) between nested models. The ORCO protein phylogenetic tree generated above using *P. siccifolium* and *C. hookeri* branches as foreground lineages was used as the tree topology imported into PAML.

### Identification of putative seminal fluid proteins (SFPs)

ORFs of transcripts over-represented in the male terminalia were identified with TransDecoder [53], a plugin from the Trinity package. Signal peptides and sub-cellular locations were identified using SignalP (v4.1) [54] and ProtComp (v9.0) (http://linux1.softberry.com), respectively.

## Results and Discussion

### *De novo* transcriptome assembly

The *C. hookeri* transcriptome constructed in the current study integrates multiple assemblies derived from five different tissues. The pooled set of approximately 113 million PE reads (Table 2) sequenced from antenna, midgut and terminalia were first quality controlled and then subjected to *de novo* assembly to construct an initial transcriptome assembly. The more than two million contigs generated from three *de novo* assemblers under the use of various K-mers were then merged together to produce a set of contigs. The leg transcriptome assembly was produced from 25,969,776 PE reads (150 bp; GenBank accession: SRR2230521) and contained 146,050 contigs. This assembly, after filtering cross-species contamination, has been deposited at DDBJ/EMBL/GenBank as a Transcriptome Shotgun Assembly project under the accession GDVG00000000 (vGDVG01000000). The Head and prothorax transcriptome was assembled from 56,112,518 single reads with the read length of 50 bp and the assembly contained 84,307 contigs (Data avalible on the Dryad database: http://datadryad.org/resource/doi:10.5061/dryad.kc826) [15].

**Table 2:** Transcriptome assembly summary.

	Raw pairs	Clean pairs
**Antenna**	14,016,766	13,614,596
**Midgut**	19,781,777	19,269,565
**Terminalia**	22,538,169	21,815,983
**Total**	56,336,712	54,700,144

The final *C. hookeri* transcriptome assembly was generated from merging the three assemblies described above to create a super-set of contigs, and then redundancy, potentially miss-assembled and non-coding transcripts were removed. This yielded a consensus transcript set comprising 77,140 contigs made up of 69.8 Mbp (Table 3) (Dryad doi:10.5061/dryad.p52sq).

**Table 3:** Summary of the transcriptome assembly.

	Contigs
**Total (bp)**	69,789,602
**Number**	77,140
**Number (> 1000 bp)**	17,117
**N50 (bp)**	1,879
**Shortest (bp)**	124
**Longest (bp)**	38,180
**Mean (bp)**	905
**Median (bp)**	407
**GC%**	46.91
**N%**	0.01

### Assessment of assembly

The quality and completeness of the combined *C. hookeri* transcript set was assessed using CEGMA by searching for the presence of highly conserved eukaryotic core orthologues. Out of the 248 core proteins, 235 (94.76%) were completely present (alignment length defined as longer than 70%) and four proteins were found with partial sequences (alignment length defined between 50% and 70%). In addition, there was an average of 2.8 orthologues per core protein and 76.2% of the CEGMA proteins detected in the *C. hookeri* transcriptome were present in multiple transcripts. Aligning raw data against the assembly revealed 92.3% of PE reads and 96.5% of head and prothorax reads mapped back to the raw assembly (assembly pre EvidentialGene pipeline). In addition, the percentage of Blast matches to fungal, plant, protist, bacterial and viral accessions was about 2.5%, indicating very low levels of xenobiotic RNA contamination in our samples. All sets of mapping results highlight that the *C. hookeri* transcriptome encompasses the majority of expressed genes present in the assembly and therefore, is a comprehensive resource for analysing genes of interest in this species.

### Gene functional annotation

More than half (56.1%) of transcripts had matching records (e-value < 10^-5^) from GenBank (*nr*). As expected, the vast majority of the Blast top-hits (83.8%) were with known arthropod proteins, from which 78.6% of the matches came from the eusocial termite *Z. nevadensis*, followed by 9.3% and 5.9% from the red flour beetle *Tribolium castaneum* and the spider *Stegodyphus mimosarum*, respectively.

**Fig 1:**
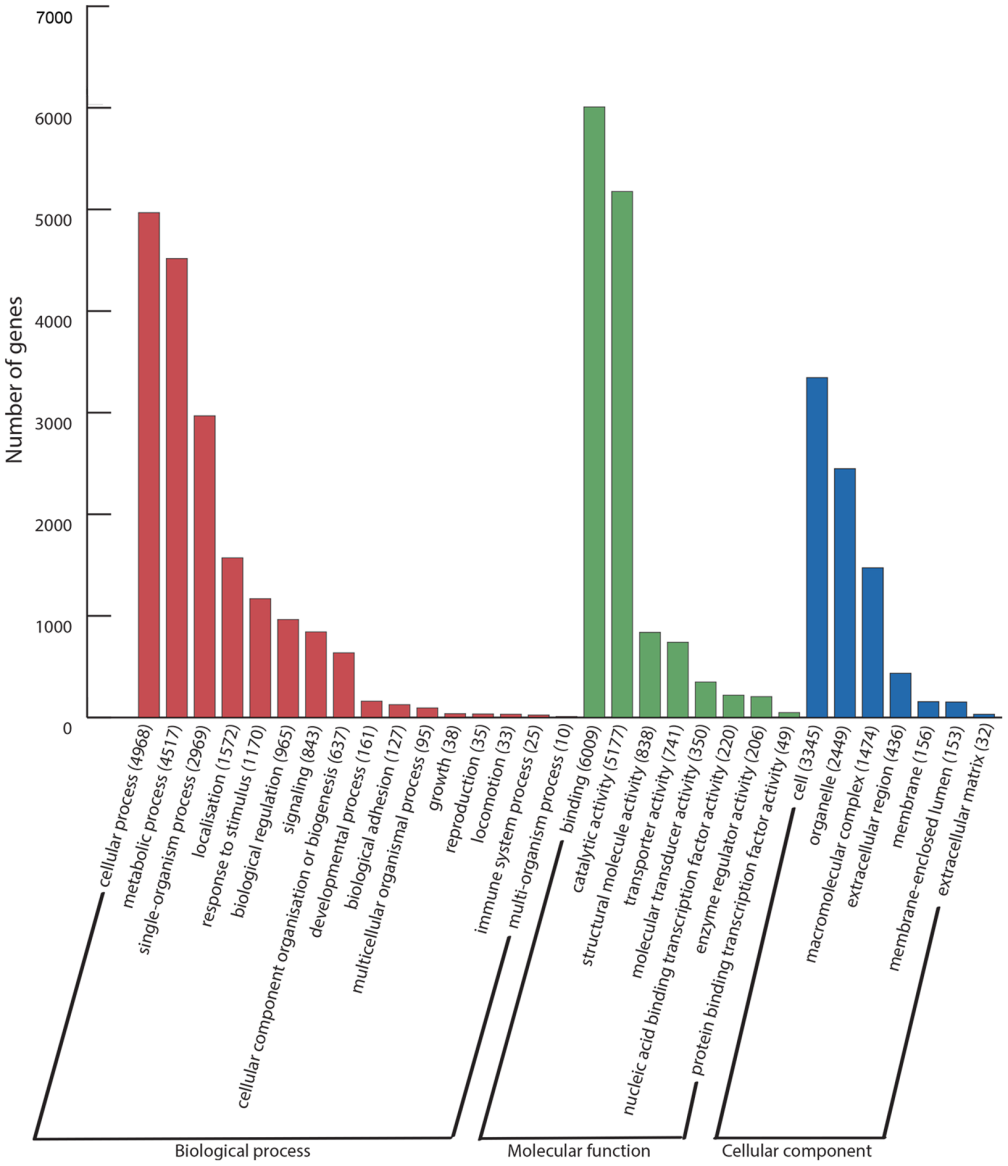
Distribution of the annotated transcripts into GO slim categories. Gene ontology terms of level 2 are shown.

Among all the contigs with significant Blast hits, 16,518 can be assigned to at least one GO term and they in turn were further refined to GO slim terms. This analysis resulted in 9,271 (12.5%), 14,905 (19.3%), and 5,586 (7.2%) of the transcripts having an assigned term from the categories of biological process (BP; GO: 0008150), molecular function (MF; GO: 0003674) and cellular component (CC; GO: 0005575), respectively (Fig 1).

### Gene expression repertoire

**Fig 2:**
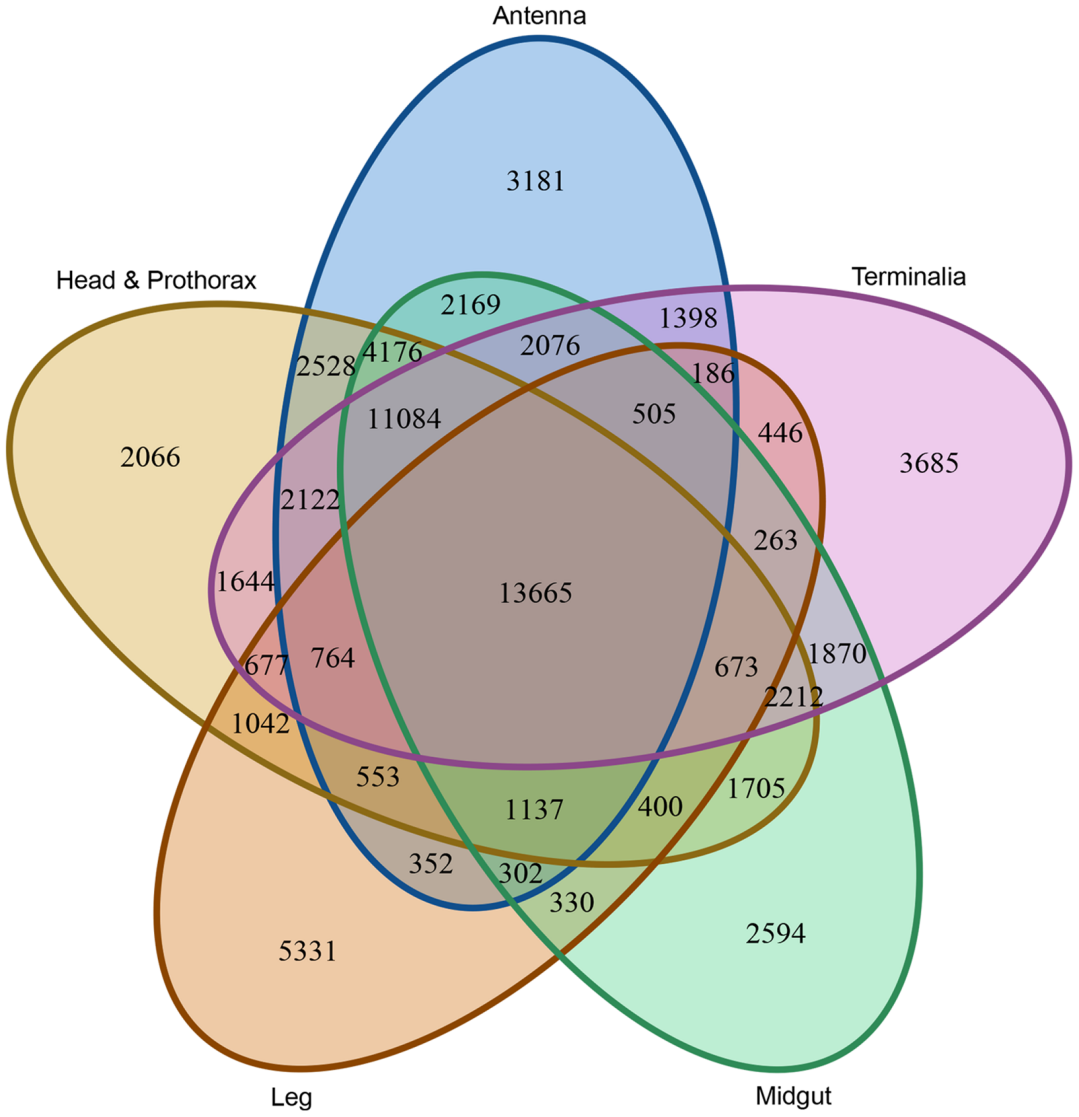
Venn diagram showing the distribution of shared and unique transcripts that were represented among the libraries of the antenna, midgut, terminalia, head and prothorax and leg. A transcript is considered as present in the library if it achieved an ECS value of larger than one. The Venn diagram was created using VennDiagram [55], an R package.

The distribution of shared and unique transcripts present in the libraries are shown in Fig 2. The analysis of the over-representation of library specific sequences focused on the antenna, midgut and terminalia. The number of transcripts over-represented in these three libraries were 1,286, 915 and 2,045, respectively, and 488, 416 and 713 of these were assigned with GO terms (S1 Table).

**Fig 3:**
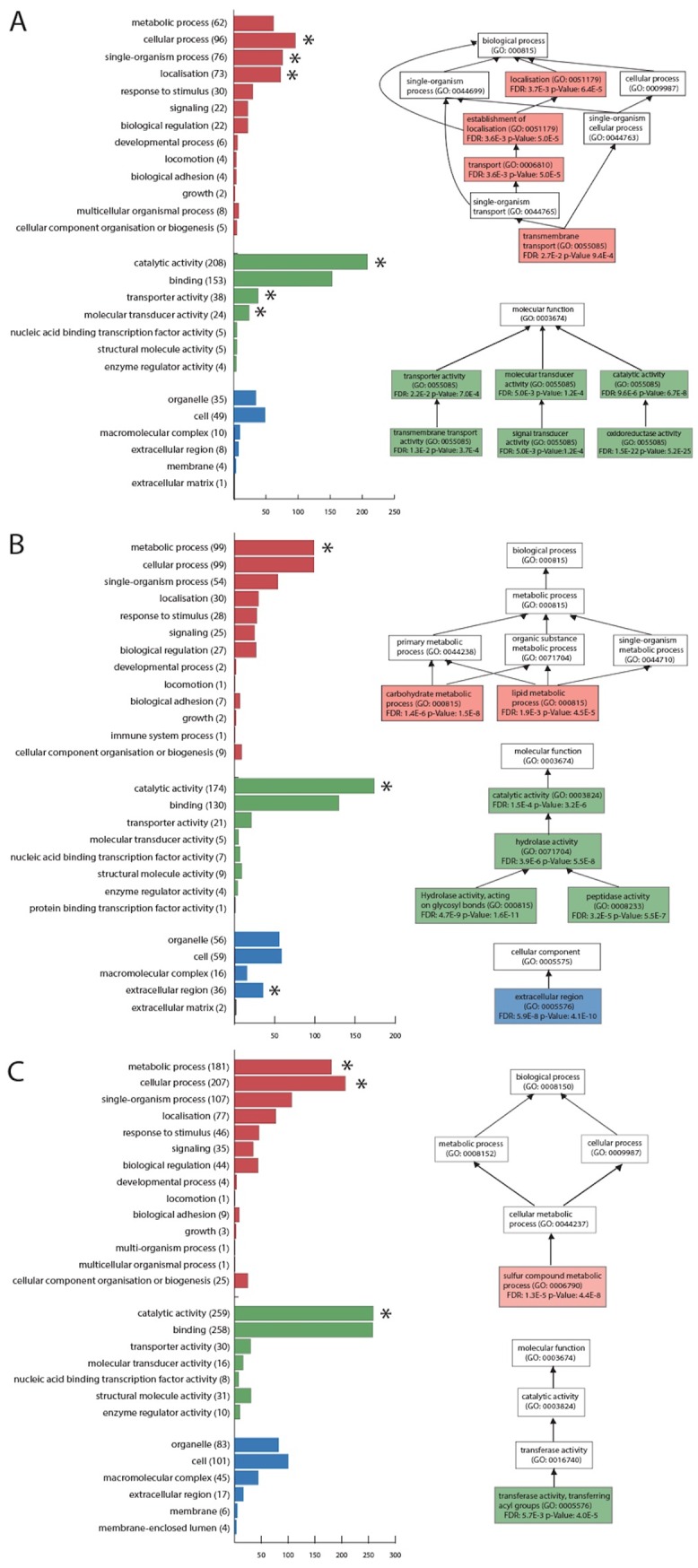
Distribution of the three GO categories in the (A) antenna, (B) midgut and (C) terminalia libraries and the significantly enriched GO terms detected from the transcripts with tissue-biased expression. *Red*, *green* and *blue* indicate the level two BP, MF and CC terms respectively, which were predicted from the transcripts showing tissue-biased expression. ‘*’ indicates the term is enriched or it contains enriched terms from higher levels. The enriched GOs and their hierarchical relationships with the lower level GOs are shown on the right side.

Distributions of transcripts with tissue-biased expression among the three tissue libraries and their enriched GO terms are given in Fig 3. Over-represented GO terms from MF category that were identified as being enriched in the antennae were ‘transmembrane transporter activity’, ‘signal transducer activity’ and ‘oxidoreductase activity’. These terms contain transcripts annotated as OBPs, ORs and odorant degrading enzymes largely associated with olfactory functions. In comparison, transcripts that were over-represented in the midgut library included many belonging to the families of glycoside and proteolytic hydrolase, resulting in the enriched GOs such as ‘hydrolase activity acting on glycosyl bonds’ and ‘peptidase activity’, corresponding to the function of intestinal digestion. The ‘transferase activity transferring acyl group’ was the only enriched MF term in the library of male terminalia. This group contains *γ*-glutamyltranspeptidase, a protease has been reported to be enriched in *Drosophila melanogaster* seminal fluid and is involved in maintaining a protective redox environment for sperm [56].

### Female olfactory gene repertoire

Olfaction plays an important role in the life of an insect, being used to detect and respond to environmental odours, thereby locating potential mates, and finding food [57]. Gene families involved in olfaction, including OBPs, Chemosensory Binding Proteins (CSPs), ORs, IRs, and in some cases GRs, have been detected across insect orders, many of which are predominantly expressed in antennae [14, 41, 58–60]. In Phasmatodea, eight OBP N-termini from the species of *E. tiaratum*, *E. calcarata*, *Acrophylla wuelfingi*, *S. sipylus* and *Carausius morosus* [61, 62], and three partial coding sequences of CSPs from *E. calcarata* [40] have been characterised. In addition, transcripts of 12 ORs, 13 IRs and three GRs from *P. siccifolium* [14] were predicted from RNA-Seq. These studies suggest phasmids have a fully developed repertoire of olfactory gene families.

#### OBP and CSP

**Fig 4:**
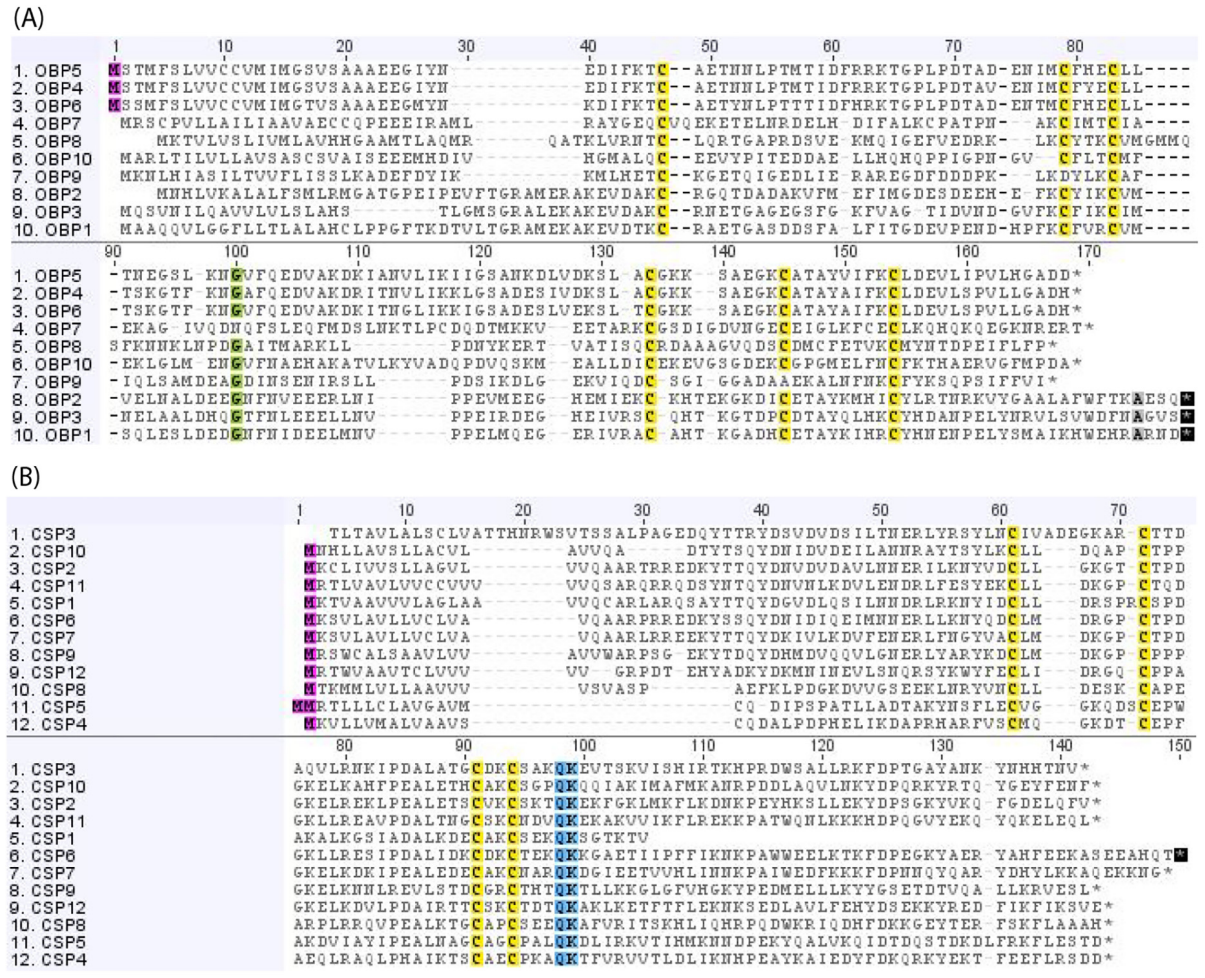
Amino acid alignments of predicted *C. hookeri* OBPs (A) and CSPs (B). Conserved cysteines are highlighted in yellow.

With high concentrations in the sensillum lymph of antennae, OBPs are small hydrophilic carrier proteins that are capable of binding small molecules [63]. This protein family is large, and contains subfamilies such as General Odorant Binding Proteins (GOBPs) and Pheromone Binding Proteins (PBPs) that specialise in carrying general odorants and sex pheromone components respectively [63–65]. CSPs are smaller carrier proteins, and similar to OBPs, they are also found in the sensillum lymph, and have been shown to bind molecules including odorants and pheromones [63]. We identified ten olfactory and 12 chemosensory binding proteins in *C. hookeri*. Sequence information and alignments showing conserved cysteines are shown in Table 4 and Fig 4, respectively.

**Table 4:** Summary of *C. hookeri* putative CSP and OBP transcripts.

Category	Transcript ID	Name	Length (bp)	Length (aa)	Hit Accession	E-value
**OBP**	Chv0TRINITY_0005088	OBP1^Φ^	653	158	KDR09909	2.68E-41
	Chv1SOAPK29_0141332	OBP2^Φ^	504	155	AAN15921	1.38E-35
	Chv1SOAPK35_0145937	OBP3^Φ^	654	150	KDR09910	6.52E-43
	Chv1VELVK45_0023357	OBP4	611	146	AID61301	2.82E-08
	Chv1VELVK45_0023358	OBP5	499	146	AID61301	9.43E-08
	Chv1VELVK45_0023359	OBP6	562	146	ABM05970	5.63E-07
	Chv1VELVK75_0000982	OBP7^Φ^	732	148	ACR39388	4.96E-09
	Chv1VELVK85_0005072	OBP8^Φ^	542	141	KDR13658	2.97E-35
	ChvKITESOAP_0129398	OBP9	410	135	AEP27187	2.11E-21
	ChvKITESOAP_0139181	OBP10^Φ^	603	145	AII00991	1.00E-11
**CSP**	Chv1SOAPK25_0127093	CSP1*	376	94	NP_001039280	1.11E-18
	Chv1SOAPK65_0069724	CSP2	783	129	AAM77025	2.00E-36
	ChvKITESOAP_0138844	CSP3*^Φ^	590	137	AEP27186	8.34E-30
	Chv0TRINITY_0001321	CSP4	821	116	AIW65103	3.07E-12
	Chv0TRINITY_0001337	CSP5	556	118	AIW65100	3.52E-12
	Chv0TRINITY_0009092	CSP6	483	135	AAM77025	4.04E-12
	Chv1SOAPK21_0133512	CSP7	762	134	AAD30552	1.57E-37
	Chv1SOAPK25_0128356	CSP8	394	122	AIW65103	1.01E-22
	Chv1SOAPK85_0010512	CSP9	545	128	AEP95756	2.12E-19
	Chv1TRINITY_0124810	CSP10	565	124	AID61322	1.41E-30
	Chv1VELVK45_0002302	CSP11	832	130	AAM77025	6.80E-30
	Chv1VELVK65_0001291	CSP12	525	127	AGZ04911	1.13E-22

“*” indicate partial sequences and “^Φ^” indicates the transcript was over-represented in the antenna library when it was compared with the other four tissue libraries.

Most OBP proteins (*OBP1*-*OBP8* and *OBP10*) contain six conserved cysteines with the spacing pattern of C_1_-X_26−29_-C_2_-X_3_-C_3_-X_41−43_-C_4_-X_8−10_-C_5_-X_8_-C_6_ (where X is any amino acid). The exception is *OBP9*, missing two conserved cysteines at positions two and five. The 12 *C. hookeri* CSPs all contain four conserved cysteines, with *CSP1* and *CSP3* missing amino acids at their 5’ and 3’ ends, respectively.

The expression level of the identified *C. hookeri* OBPs varied significantly among tissues (S1 Table). Analysis showed six OBPs (*OBP1*, *OBP2*, *OBP3*, *OBP7*, *OBP8*, and *OBP10*) and one CSP (*CSP3*) were over-represented in the female antenna library. Among these, *OBP2*, *OBP3*, and *OBP8* were most likely to be antennae-specific, as they were very lowly expressed in every other tissue library (FPKM < 1). In contrast, *OBP1*, *OBP7*, and *OBP10* were detected in antennae, head and prothorax, and *OBP1* and *OBP10* were also expressed in the composite leg.

**Fig 5:**
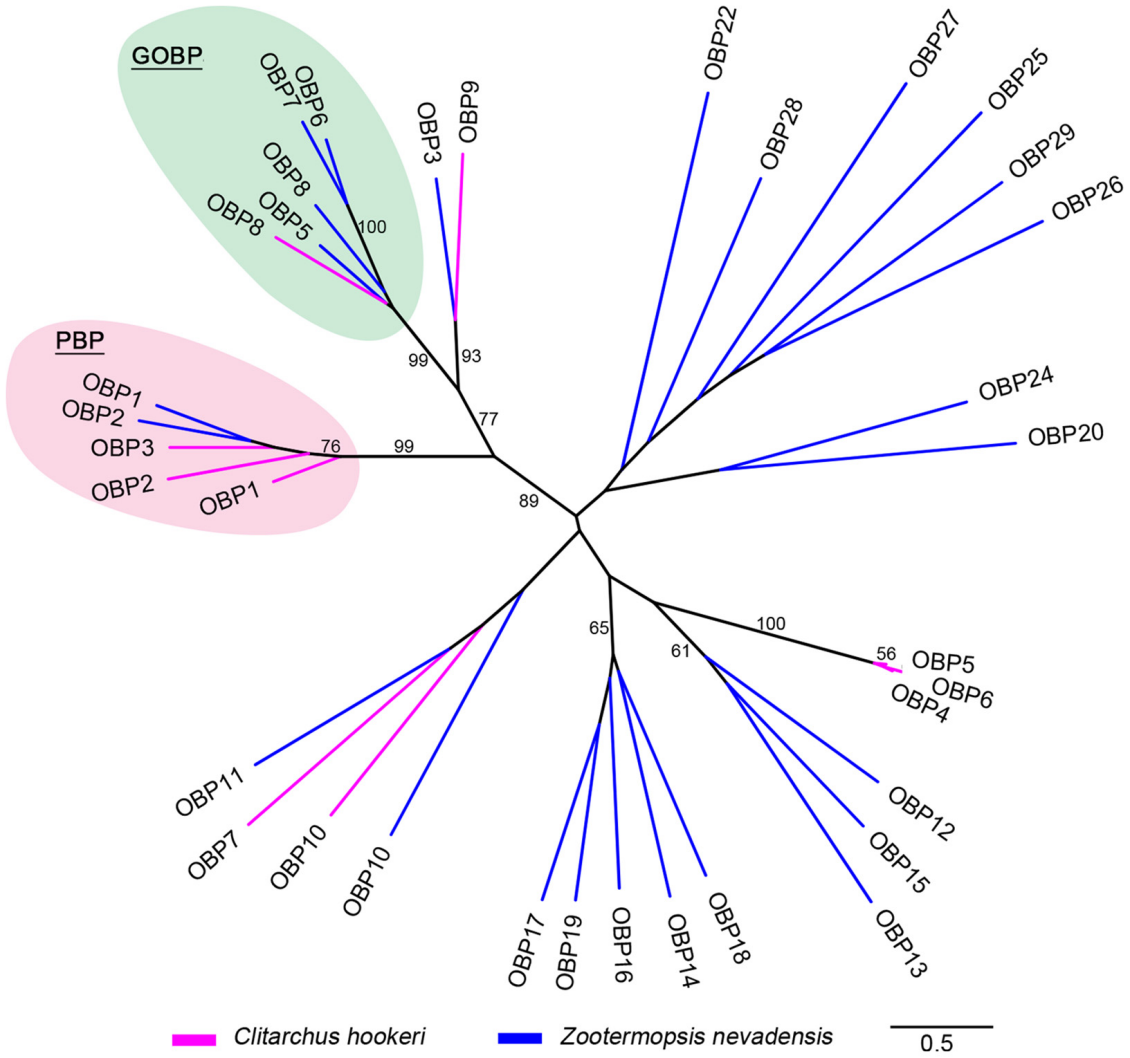
Phylogenetic tree for *C. hookeri* and *Z. nevadensis* OBP protein sequences. The accession IDs of the sequences downloaded from GenBank that are used for this phylogeny are in S3 Text. The other *Z. nevadensis* OBP sequences from the same species were obtained from the supplementary material of Terrapon et al. [39]. Pink and blue branches represent *C. hookeri* and *Z. nevadensis* OBPs respectively. The bootstrap values larger than 50 are written next to each branch. The potential PBP and GOBP groups from the two species are circled with light pink and light blue respectively. The scale bar represents branch lengths.

*OBP1* was the most abundant OBP in the *C. hookeri* female antennae (FPKM: 26,978.3), and had a highest scoring blast match to a *Z. nevadensis* PBP (Table 4). In the phylogeny, *OBP1* is closely clustered with the two *Z. nevadensis* PBPs (*OBP1* and *OBP2*) (Fig 5), indicating the *C. hookeri* *OBP1* is most likely a PBP. A recent study has demonstrated that male *C. hookeri* detects females using airborne chemical cues, most likely pheromones emitted by the females [7]. The presence of a higher density of basiconic sensilla on male than female antennae further indicates that males are responsible for long range pheromone reception [7]. Therefore, a high abundance of a PBP transcript in a *C. hookeri* female antennae seems unexpected. Interestingly, a PBP (N-terminal amino acids) has been also detected in the antennae of female *C. morosus* [62], an obligate parthenogenetic stick insect species, suggesting that the presence of the PBP was not associated with mate searching. Rather, it might be useful for females to space themselves out in the environment through detecting at least some components of sex-pheromones released from other females [66] or it could be utilised in sensing a subset of plant volatiles [67]. Furthermore, *OBP1*, *OBP2* and *OBP3* were shown to cluster phylogenetically with the two *Z. nevadensis* PBPs (Fig 5), indicating the possible presence of multiple PBPs in female *C. hookeri*. We also found *C. hookeri* *OBP8* was nested in the *Z. nevadensis* *GOBP19a* clade (*OBP5*—*OBP8* with branches coloured by blue in Fig 5), suggesting that the protein is a general odorant binding protein.

OBPs containing less than six conserved cysteines are referred to a subgroup named non-classic Minus-C [68]. These proteins have been demonstrated to exist in the genomes of a range of insect species, such as *Ceratitis capitata*, *D. melanogaster*, *Tenebrio molitor*, and *Anopheles gambiae*, with functions always inferred as non-olfaction due to the fact that the majority are actually more highly expressed in non-olfactory organs, such as male accessory gland and salivary gland [69–71]. As a member of Minus-C subgroup, *C. hookeri* *OBP9* was highly abundant in the male terminalia library (FPKM: 1,377.6), but its expression was also detected in the female antenna (FPKM: 299.8), leg (FPKM: 15.4) and head and prothorax (FPKM: 2.1), suggesting that this protein might be involved in multiple biological processes. The predominant expression in the male accessory gland suggests that *OBP9* plays a role in sexual reproduction that is likely to improve offspring survival. It has been reported that an OBP detected in the seminal fluid of *Helicoverpa armigera* and *H. assulta* can be transferred to females through mating, eventually being found on the surface of fertilised eggs [72]. The presence of this protein was suggested to favour the spreading of the eggs to alleviate cannibalism among larvae [72]. Phylogenetic analysis shows *OBP9* is closely related to both the PBP and GOBP clades, suggesting potential functions involved in the detection of pheromone components or general odorants (Fig 5).

*OBP5*, *OBP6*, and *OBP7*, found to be adjacent to each other on one genomic scaffold (scaffold1029; Dryad database: http://datadryad.org/resource/doi:10.5061/dryad.p52sq), are present as paralogues forming a single clade in the phylogeny (Fig 5). Their high sequence similarity (83–89% amino acid similarity) may lead to spurious estimates of the expression level based on RNA-Seq methods because it is possible that the reads derived from one of the genes could have aligned with one of the other two. However, all of these genes appear to be transcribed at much lower levels than most of the other OBPs. *OBP5* and *OBP6* show expression in the head and prothorax library (FPKM: 24.3 and 63.5) and *OBP6* may also be expressed in antennae (FPKM: 8.2) and leg (10.6), whereas *OBP4* was only detected with a small number of transcripts from the leg library (FPKM: 6.7). Phylogenetic analysis also shows these three proteins form a single clade more closely related to *Z. nevadensis* *OBP12*, *OBP13* and *OBP15*. It is unknown which type of molecules these OBPs might be able to detect, but their recent duplication indicates they may underlie key adaptive events in the evolution of *C. hookeri*. However, low expression raises the possibility that all or some of these genes are non-functional, such as pseudogenes.

All of the identified CSPs were found to be present, but not over-represented in the antennae, except for *CSP3*, indicating they may also function as general carriers across multiple tissue types. *CSP6* was over-expressed in male terminalia (FPKM: 83.1), indicating that it potentially plays a role in sexual reproduction, while *CSP10* was over-expressed in midgut (FPKM: 9729.6), most likely functioning as a carrier in digestion.

#### OR, IR, SNMP, and GR

**Fig 6:**
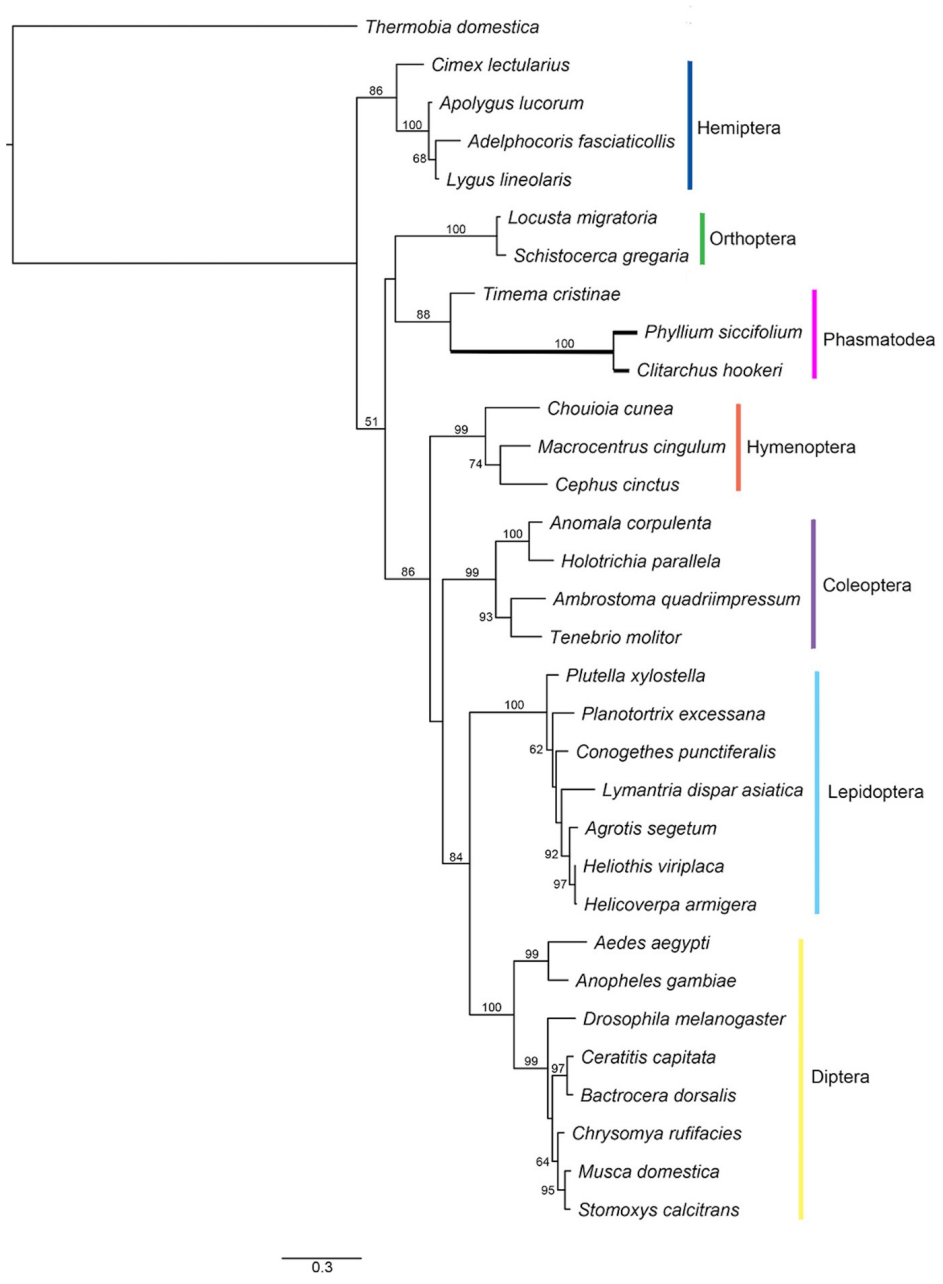
Phylogenetic tree of ORCOs. The coloured vertical bars indicate the sequence clades corresponding to the insect orders. The subclade branches containing *P. siccifolium* and *C. hookeri* are in bold. Scale bar represents branch length. The accession IDs of the sequences used are in S3 Text.

ORs and IRs present on the dendrites of olfactory sensory neurons detect a range of odorants shuttled by OBPs and CSPs from the environment, the activation of which leads to ion channel activation and receptor neuron depolarization [73–75]. The identified *C. hookeri* putative protein sequences of these genes are shown in S1 Text. Of the 16 ORs, four (*OR2*, *OR5*, *OR11* and *OR16*) are likely to encode full-length proteins. *OR16* is annotated as an odorant receptor co-receptor (ORCO), a highly conserved receptor across insects that is required for ion channel function and dendritic localisation of other ORs [76]. *Timema cristinae* ORCO was identified by performing blast searches using *C. hookeri* amino acid sequence against the genome (downloaded from http://nosil-lab.group.shef.ac.uk/?page_id=25). A phylogenetic tree of ORCO amino acid sequences shows *C. hookeri* is nested within Phasmatodea, grouping with another phasmid *P. siccifolium*, which in turn is closely related to Orthoptera (Fig 6). The branch leading to *C. hookeri* and *P. siccifolium* is considerably longer than the other branches, suggesting an accelerated rate of evolution. However, a branch selection test implemented in CODEML (from PAML (v4.8) [52]) did not show evidence of positive selection on this branch (*p* = 1). The *C. hookeri* IRs have higher sequence similarity to homologous IRs identified in *Z. nevadensis* compared to the homologous ORs identified in the two genomes (e-values in Table 5). This finding suggests the ORs likely evolve faster than the IRs in phasmids. Apart from five IRs likely to encode full-length proteins, the others only contain partial coding sequences.

**Table 5:** Summary of *C. hookeri* putative OR, IR and SNMP OBP transcripts.

Category	Transcript ID	Name	Length (bp)	Length (aa)	Hit Accession	E-value
**OR**	Chv1SOAPK35_0009687	OR1*	532	168	XP_001120660	1.23E-10
	Chv1VELVK29_0021980	OR2	1567	448	KDR14144	2.16E-21
	Chv1VELVK29_0015825	OR3*	630	134	EFN78840	2.96E-14
	Chv1VELVK29_0033146	OR4*	404	106	XP_008207129	1.04E-07
	Chv1VELVK35_0019903	OR5^Φ^	1722	452	XP_011166508	1.77E-14
	Chv1VELVK45_0034664	OR6*	594	151	AII01061	4.16E-24
	Chv1VELVK21_0035864	OR7*^Φ^	1072	292	BAG12817	1.99E-14
	Chv1VELVK21_0058449	OR8*^Φ^	2466	388	KDR17467	1.56E-17
	Chv1VELVK21_0062558	OR9*^Φ^	951	259	KDR14144	1.65E-21
	Chv1VELVK21_0073095	OR10*	719	193	KDR14144	9.37E-20
	Chv1VELVK21_0077746	OR11^Φ^	1533	424	KDR14144	9.20E-16
	Chv1VELVK21_0088043	OR12*	993	291	KDR11214	8.44E-18
	Chv1VELVK25_0038317	OR13*	941	226	KDR16425	2.03E-21
	Chv1VELVK35_0042637	OR14*	880	233	AEE62637	9.90E-21
	Chv1VELVK25_0067528	OR15*	573	146	KDR14144	4.20E-21
	Chv1VELVK21_0029367	OR16^Φ^	3087	477	KDR12002	3.11E-180
**IR**	Chv1SOAPK25_0135053	IR1*	538	178	AFC91761	4.29E-44
	Chv1VELVK21_0063588	IR2*^Φ^	1092	309	AII01119	1.19E-76
	Chv1VELVK25_0038786	IR3*	618	205	AHA80144	3.05E-47
	Chv1VELVK21_0041817	IR4*^Φ^	2156	606	AHA80144	0
	Chv1VELVK21_0061997	IR5	1992	644	XP_004932732	9.08E-179
	Chv1VELVK21_0029672	IR6*	937	281	XP_001655464	4.70E-99
	Chv1VELVK25_0009676	IR7	2419	446	XP_001661131	6.39E-23
	Chv1VELVK65_0046897	IR8	2731	838	XP_008201635	0
	Chv1VELVK21_0053197	IR9*^Φ^	2216	618	KDR23344	9.30E-151
	Chv1VELVK21_0109764	IR10*	722	234	KDR23343	6.40E-53
	Chv1VELVK25_0018305	IR11*	3370	386	XP_002431127	1.97E-101
	Chv1VELVK25_0043548	IR12*^Φ^	3173	299	KDR23344	3.19E-51
	Chv1VELVK55_0002278	IR13	2316	614	KDR16875	2.17E-41
	Chv1VELVK21_0047941	IR14^Φ^	2810	639	KDR14986	1.26E-163
	Chv1VELVK21_0052279	IR15^Φ^	2262	598	KDR23343	6.69E-142
	Chv1VELVK21_0091436	IR16*	1720	515	KDR24341	6.42E-176
	Chv1VELVK45_0004605	IR17*^Φ^	1357	335	KDR22647	2.09E-64
**GR**	Chv1SOAPK35_0133587	GR*	434	108	KDR10758	5.16E-14
**SNMP**	Chv1VELVK55_0003575	SNMP	5437	691	XP_011195945	4.55E-89

“*” indicate partial sequences and “^Φ^” indicates the transcript was over-represented in the antenna library when it was compared with the other four tissue libraries.

Transcript abundance analysis showed six ORs (*OR5*, *OR7*, *OR8*, *OR9*, *OR11* and *OR16*) and seven IRs (*IR2*, *IR4*, *IR9*, *IR12*, *IR14*, *IR15* and *IR17*) were over-represented in the antennae library, with expression levels significantly lower than the OBPs and CSPs. Except for the antennae, short reads that were aligned to the putative OR transcripts and most of the IR transcripts from the other four tissue libraries were rare, indicating the main function of the proteins encoded by these transcripts is olfactory reception. *IR11* and *IR13* were shown to be expressed in all tissue libraries indicating they are likely to be involved in multiple roles. *IR6* and *IR7* were over-expressed in head and prothorax and male terminalia, respectively.

In addition to the two major receptor families described above that are essential for olfaction, other proteins such as sensory neuron membrane protein (SNMP) and GR may also play a role. SNMP is suggested to act in concert with ORs to capture pheromone molecules in *D. melanogaster* and the moth *Heliothis virescens* [64, 77, 78], while GR is known to be associated with taste [79]. A complete *C. hookeri* SNMP (Table 5) encoding 691 amino acids was identified, which had pairwise similarities of 24.7% and 25.4% with the two moth SNMPs, respectively. The expression of this *C. hookeri* SNMP was observed in all tissues, with the most transcripts coming from the midgut (FPKM: 65.9). A partial *C. hookeri* GR transcript encoding 108 amino acids with the highest scoring blast alignment (non-hypothetical protein) of a *Pediculus humanus corporis* GR (GenBank:XP_002426236; e-value = 2.73e-14) was also identified. The sequence information of these two transcripts are shown in Table 5 and S1 Text.

The numbers of putative *C. hookeri* OR, IR and GR are comparable to the repertoire of these gene families in *P. siccifolium* [14], with slightly higher numbers of OR and IR occurring in *C. hookeri*. It is possible that OR and IR have undergone multiple gene duplications in *C. hookeri*; however, in the absence of a complete genome sequence, it is difficult to determine if we have obtained the total number of family members. Also, the *C. hookeri* transcriptome assembly only contains transcripts derived from female antennae, while potential male-biased ORs and IRs, such as *OR7* and *OR6* in *E. postvittana* [41], may be missing. Finally, sequencing to a greater depth may identify further ORs.

In summary, the antennae showed expression of the majority of expected olfactory proteins. The total number of identified *C. hookeri* olfactory genes from our transcriptome are generally lower than the number of genes identified from the genomes of *Locusta migratoria*, *Acyrthosiphon pisum*, *T. castaneum*, and *Bombyx mori* (Fig 4a from [80]). Only IRs are more numerous than the number present in *L. migratoria* and *A. pisum*.

### Digestive enzymes

#### Endogenous cellulase

Sequencing the *C. hookeri* midgut transcriptome enables us to examine its digestive enzymes, especially GH9 endogenous cellulase, which has previously been found to have undergone gene copy expansion [5] and have divergent functions in phasmids, such as being capable of breaking down xylan, xyloglucan, and/or glucomannan [18]. The first insect gene from GH9, also called endo-beta-1,4-glucanase (EG), was initially found in a termite, and is now believed to have existed in the ancestor of all metazoans [81, 82]. High GH9 activity in the anterior midgut of stick insects has been recently confirmed in both *E. calcarata* and *Entoria okinawaensis* [6], while the prediction of two to seven cellulase transcripts from the midgut transcriptomes of six phasmid species, respectively, indicate the presence of multiple gene copies in Phasmatodea genomes [5].

We identified five putative *C. hookeri* EG transcripts, named *EG1* to *EG5*, with similarity to the termite and cockroach EG proteins (percentage amino acid identity between 56.5% and 66.6%). These transcripts contain Pfam GH9 protein domains and show moderately high similarity (> 57%) to known EG coding sequences on Genbank (*nt*). A comparison between these transcripts and the preliminary *C. hookeri* genome assembly indicates all the putative EG proteins are encoded from five gene copies. As expected, these cellulase genes show over-expression in the female midgut, suggesting they function in *C. hookeri* intestinal digestion and most likely, like other phasmids, play a role in degrading multiple plant cell wall components into sugar monomers [18].

**Fig 7:**
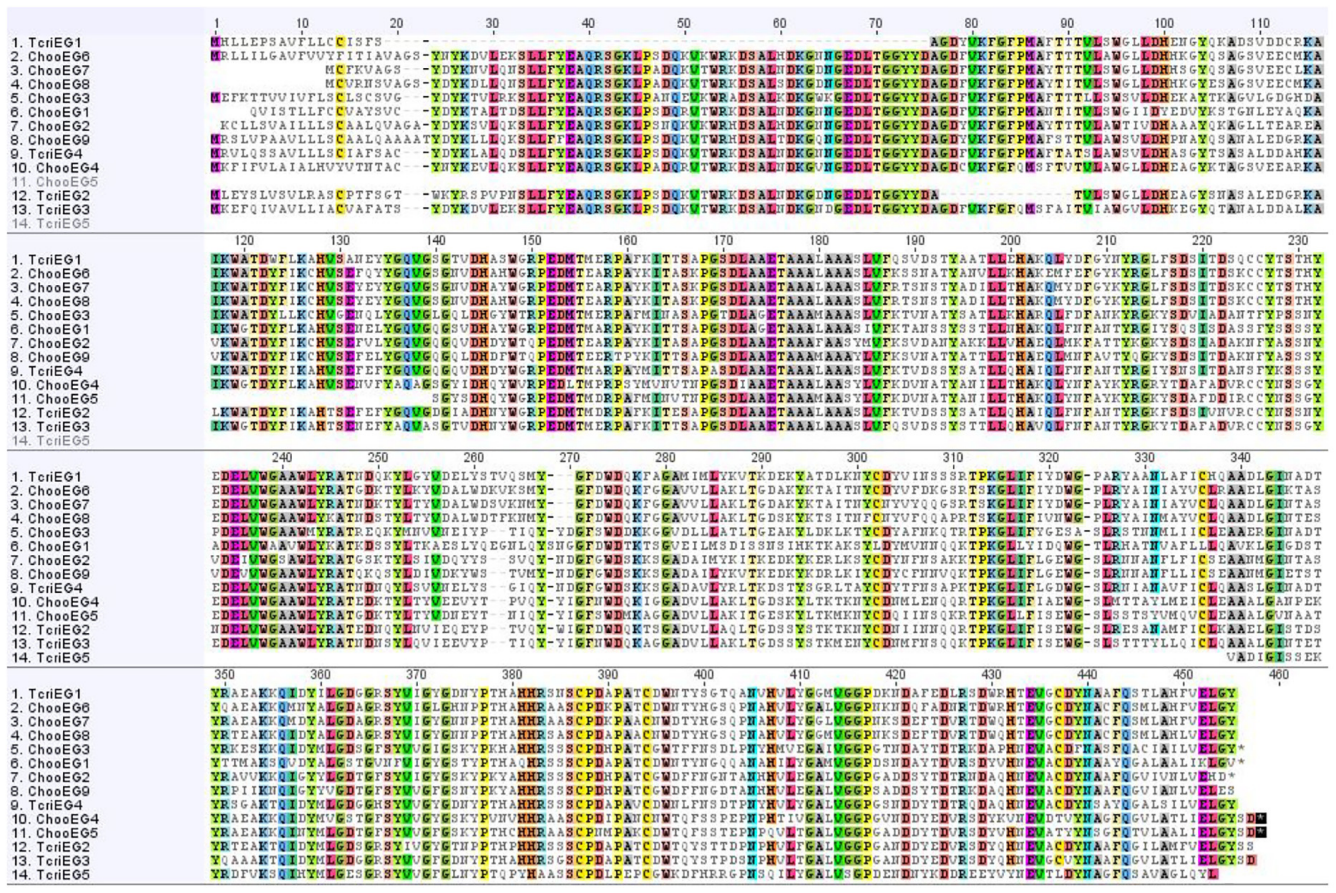
Amino acid alignments of predicted *C. hookeri* and *T. cristinae* EGs. The conserved regions are highlighted with colours.

Surprisingly, we also found four additional cellulase copies encoding full-length protein sequences present on the same genomic scaffold that contains *EG2*, *EG4* and *EG5* (Table 6). Three of these, *EG6*, *EG7*, and *EG8*, show pairwise amino acid identities between 84% and 92%, significantly higher than those between any two of the other putative EGs, indicating a recent duplication. The protein sequence of the other gene, *EG9*, had 80.4% identity to *EG2*. Homology searches using these EG coding sequences against both *C. hookeri* raw and final transcriptome assemblies did not yield any similar hits, indicating they were most likely absent from the transcriptome data. There are several possibilities that might lead to the absence of these EGs in our *C. hookeri* midgut transcriptome. First, the genes are silenced or transcribed at extremely low levels and could not be captured by RNA-Seq with the sequencing depth conducted by our study. Second, the construction of an RNA-Seq library may introduce library bias that leads to the false negatives. Third, the genes may be transcribed in different tissues of the body rather than midgut. It is also possible that some of them are pseudogenes, thereby lacking expression. Overall, we conclude that there are at least nine cellulase gene duplicates of ORFs present within the *C. hookeri* genome, with a minimum of five expressed in the midgut of female adults (Table 6 and Fig 7). This estimated copy number of GH9 cellulases is higher than the total number of its transcripts/genes detected from other insect lineages [5, 81, 83], indicating a complex evolutionary history of GH9 in stick insects.

**Table 6:** Summary of *C. hookeri* and *T. cristinae* putative GH9 genes.

Transcript ID or Genomic position	Name	Length (bp)	Length (aa)	Hit Accession	E-value
Chv1VELVK35_0068512	ChooEG1*^Φ^	1344	448	BAA34050	0
Chv1VELVK29_0092141	ChooEG2*^Φ^	1347	449	CAD54729	0
Chv1VELVK21_0117909	ChooEG3^Φ^	1347	449	AFD33365	0
Chv1VELVK21_0101553	ChooEG4^Φ^	1353	451	AAF80584	0
Chv1VELVK21_0022349	ChooEG5*^Φ^	945	315	CAD54729	1.55E-166
Scaffold1797 (8 exons: 75383-57750 bp)	ChooEG6	1353	451	CAD54729	0
Scaffold1797 (8 exons: 111023-85577 bp)	ChooEG7	1311	437	CAD54728	0
Scaffold1797 (9 exons: 157301-132988 bp)	ChooEG8	1317	439	CAD54728	0
Scaffold1797 (8 exons: 280626-265133 bp)	ChooEG9	1356	452	CAD54729	0
Scaffold24 (8 exons: 453210-441732 bp)	TcriEG1*	1185	395	CAD54730	9.82E-166
Scaffold24 (9 exons: 468621-458748 bp)	TcriEG2*	1311	437	CAD54730	0
Scaffold24 (9 exons: 481252-473897 bp)	TcriEG3	1353	451	AAF80584	0
Scaffold24 (9 exons: 489799-500978 bp)	TcriEG4	1347	449	CAD54728	0
Scaffold551 (299934-288976 bp)	TcriEG5*	381	127	XP_002426465	44E-50

“*” indicate partial sequences and “^Φ^” indicates the transcript was over-represented in the antenna library when it was compared with the other four tissue libraries. The nucleotide length in this table indicates the length of ORF predicted from either the transcript if available or the genomic scaffolds (Dryad: doi:10.5061/dryad.p52sq).

EG sequences of the stick insect *T. cristinae*, sister group to all other phasmids, were identified by the use of the *C. hookeri* amino acid sequences to search against the *T. cristinae* genome. Four EGs were found from one scaffold and an additional partial sequence was detected on a different genomic sequence, bringing the total of *T. cristinae* EGs to five (Table 6). Compared with nine *C. hookeri* gene copies, this suggests that there has been a dramatic GH9 family expansion during the evolution of phasmids.

Symbiont-independent degradation of cellulose in phasmids could have selected for gene duplications within the endogenous cellulase gene family. It has been suggested that phasmid digestion is most likely to be symbiont-independent, although molecular evidence for this is restricted to the species of *R. artemis* and *P. schultei* [17, 84]. Without the assistance of symbionts, stick insects must maintain the ability to digest cellulose independently, such as secreting their own cellulase to break down plant cell walls, and this digestive efficiency might have increased through evolving more gene copies. Having additional copies of GH9 cellulase genes may increase dosage and overall expression of these genes and enhance the efficiency of cellulose digestion without relying on enzymes produced by symbionts. Additionally, duplicated gene copies could have evolved to acquire additional functions to facilitate the digestion of other polysaccharides or oligosaccharides present in plant cell walls. On the other hand, it is also possible that some of the duplicated cellulase genes have evolved different functions and are expressed in other tissues or organs [85].

**Fig 8:**
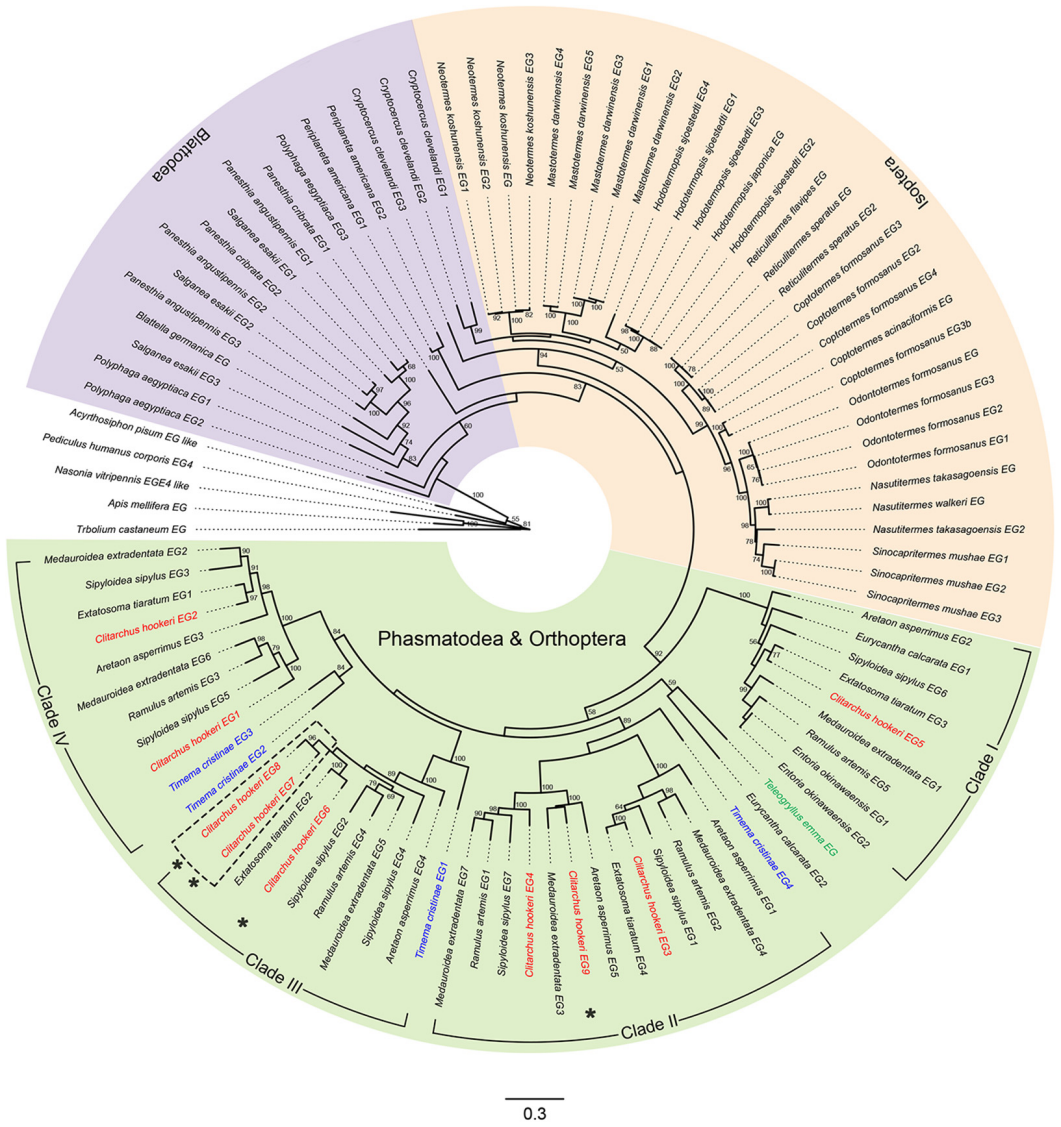
Phylogenetic tree of GH9. Maximum likelihood tree for insect derived GH9 family nucleotide sequences. Nodes are annotated with species and GH9 paralogue numbers that were either obtained from NCBI or given in this study. Species belonging to Phasmatodea and Orthoptera clade, Isoptera clade and Blattodea clade are coloured with backgrounds in light green, yellow and purple respectively. The branch labels of *C. hookeri*, *T. cristinae* and *Teleogryllus emma* sequences are in red, blue and green respectively. Dashed box highlights the two *C. hookeri* specific cellulase lineages. The four *C. hookeri* cellulases identified from the genome assembly are marked with ‘*’. Numbers above branches are bootstrap proportions and values above 50 are shown. Scale bar represents branch lengths. The accession IDs of the sequences that are used for this phylogeny are in S3 Text.

To investigate evolutionary relationships among *C. hookeri* GH9 genes and their orthologues, a maximum likelihood phylogeny was built from coding sequences (CDS) collected from a variety of insect species (Fig 8). The phylogenetic tree shows *C. hookeri* cellulases are closely related to those derived from other phasmids. The stick insect and cricket clade is nested within the cockroach clade, which is sister to a large clade containing predominantly termite sequences. In addition, phasmid sequences exhibit greater divergence among one another compared with those present in any termite and most cockroaches. Their gene duplication events occurred much earlier in the evolution of the stick insects comparing with the termite and the majority of the cockroach clades, and might be even earlier than the divergence of Phasmatodea from other insects because the cricket (*Teleogryllus emma*) sequence is nested within the phasmid clade. The nine *C. hookeri* EGs fall into four sub-clades, with each of them rooted by at least one *T. cristinae* sequence, except for clade I. We did not include *T. cristinae* *EG5* in the phylogeny because the identified sequence was only about one third of the length of other full-length sequences. However, preliminary analysis suggests this gene is likely to be clustered into clade IV. Clade I contains *C. hookeri* *EG5* as sister to the rest of clades and clade II is sister to clades III and IV. Except for clade I, each of the other clades contains two to three *C. hookeri* duplicates, whereas only clade IV contains at least two *T. cristinae* EG paralogues, which likely duplicated after the divergence of *T. cristinae* from the rest of phasmids. Clade III contains two *C. hookeri* EG paralogues (EG7 and EG8) which likely duplicated in the *C. hookeri* lineage. Those *C. hookeri* EGs that were not detected with expression in midgut all fall into the clade III, with the exception of *EG9*, which falls into a sub-clade of the clade II. The future discovery of more cellulase gene copies from other stick insects may reshape this phylogeny and reveal a different pattern of gene family evolution.

The EG transcripts identified from the study of six phasmid midgut transcriptomes [5], together with the nine and five genes identified from *C. hookeri* and *T. cristinae* in this study suggest gene gain or loss events are common occurrence during phasmid evolution. However, the absence of complete genomes makes it difficult to fully address this question. Further studies to characterise the expression patterns of all nine cellulases in *C. hookeri*, and confirmation of the cellulase gene copy number within other stick insect lineages may shed light on the evolutionary history of this essential plant cell wall digestive enzyme system in phasmids.

#### Other enzymes

Consistent with the findings from other stick insect species [5], pectinase transcripts (GH28) with the highest scoring blast alignment of *γ*-proteobacteria sequences were also present in the *C. hookeri* midgut library (S1 Table). These enzymes hydrolyse glycosidic bonds in pectin and are essential to plant pathogenic and saprotrophic fungi [86]. It has been found that, similar to beetle pectinases [87], pectinase transcripts present in phasmid midgut transcriptomes are endogenic and stick insects have obtained the genes through horizontal transfer from bacteria [8]. Comparison between the pectinase transcripts and the *C. hookeri* genome assembly reveals at least three genomic scaffolds (scaffold7807, scaffold1326 and scaffold5845) harbour pectinase-like genes (results not shown). However, none of these genes contains an intron to support the contention that these genes are true genome integrations. Nonetheless, two of these scaffolds (scaffold1326 and scaffold5845) harbour genes annotated to Arthropoda proteins, including those having the highest scoring blast alignments to the proteins of the species *Z. nevadensis*, *Solenopsis invicta*, *Haplopelma schmidti*, *Tribolium castaneum*, and *Megachile rotundata*. This may indicate that the *C. hookeri* pectinase genes are indeed endogenous. Future work involving sequencing of genomic regions containing a predicted pectinase gene contiguous with another *C. hookeri* gene will rule out potential mis-assemblies and confirm this finding.

The midgut over-represented transcripts also included glycosidases belonging to other gene families, such as *β*-galactosidase, chitinase, *β*-hexosaminidase subunit beta and glucosylceramidase, *α*-L-fucosidase, mannosyl-oligosaccharide1, 2-*α*-mannosidase, maltase, and trehalase (S1 Table). The expression of chitinase indicates that *C. hookeri* may have the ability to recycle chitinous cell walls of fungi as carbohydrate and nitrogen resources, similar to the wood-feeding beetle *A. glabripennis* [88]. Furthermore, the midgut over-expressed maltase (S1 Table), which hydrolyses maltose to the simple sugar glucose, and, together with the presence of an amylase in the transcriptome raises the prospect that *C. hookeri* is likely to be able to use starch as an energy source.

### Seminal fluid proteins

Insect seminal fluid proteins (SFPs) are a group of secreted proteins produced from male reproductive tract tissues, predominantly accessory glands, and stored in the accessory gland lumen until required. These proteins are particularly interesting for the study of sexual reproduction because they are essential to male reproductive success and their rapid evolution likely contributes to speciation [89, 90]. The specific functions of these proteins is that they are transferred to females with sperm during mating and are then involved in regulating female reproductive functions, such as inducing antimicrobial activities, increasing ovulation and egg-laying rate, and reducing the likelihood of re-mating [91]. Hitherto, proteins identified from insect seminal fluids include proteases and protease inhibitors, lipases, oxidative stress proteins, immune proteins and cell adhesion-related proteins [56, 92, 93], but none of which have been investigated in phasmids to date.

From the transcripts that were over-represented in the library of *C. hookeri* male terminalia, we predicted 711 candidate SFP transcripts by searching for signal peptides and their protein localisations in the plasma membrane or extracellularly (S1 Table). Of these, 140 had no Blast hits, indicating they might be novel proteins or proteins highly diverged in phasmids. There are a total of 28 *C. hookeri* transcripts classified with GO terms of peptidase activity and peptidase regulator activity (S2 Text), two groups of proteins that are believed to be essential and regulate reproduction through proteolytic cascades [94]. The predicted members of 22 proteases/peptidases included serine protease (including trypsin), furin protease, carboxypeptidase, *γ*-glutamyl transpeptidase, membrane metallo-endopeptidase, signal peptidase complex subunit 2, angiotensin converting enzyme (ACE) and thrombospondin type-1, and six protease inhibitors (Table 7).

**Table 7:** Summary of terminalia-enriched proteases or peptidases and protease inhibitors.

Name	Transcripts ID	Length (bp)	Length (aa)	FPKM	Hit Accession	E-value
**Serine protease**	Chv1VELVK55_0057832	1971	261	216.5	XP_001121121	2.85E-55
	Chv1VELVK35_0071739	1241	203	6.88	XP_001121121	6.22E-42
	Chv1VELVK45_0064057	1240	260	21.16	XP_317172	7.24E-57
	Chv1VELVK29_0052877	2167	285	22.71	ACX54054	9.2E-110
**Aminopeptidase**	Chv1VELVK21_0040553	1990	548	122.9	KDR21177	0
	Chv1VELVK25_0028117	1644	359	3.23	KDR21177	5.02E-81
	Chv1VELVK25_0028115	1901	514	9.1	KDR21177	1.8E-168
**MME**	Chv1VELVK55_0007423	2731	765	108.49	KDR18716	0
	Chv1VELVK21_0124071	3503	797	5.7	KDR09552	0
**GGT**	Chv1VELVK21_0017697	2375	695	3.12	XP_974407	0
	Chv1VELVK85_0006040	1907	580	189.19	KDR15762	0
	Chv1VELVK75_0011772	1948	597	85.17	KDR15762	0
	Chv1VELVK25_0015659	3680	942	0.51	KDR18594	0
	Chv1VELVK21_0024934	2697	252	2.95	KDR15762	1.7E-125
	Chv1VELVK21_0024932	6370	235	5.56	KDR15762	1.06E-84
**FURIN**	Chv1VELVK45_0035041	2290	632	1.23	XP_008545163	2.44E-07
	Chv1VELVK21_0083474	4574	1029	1.29	XP_002422846	3.05E-41
**Carboxypeptidase**	Chv1VELVK21_0032458	2769	547	22.71	KDR18068	0
	Chv1VELVK65_0051556	1517	445	1365.21	AEI58649	9.4E-105
	Chv1VELVK65_0051554	1208	342	24.07	AFZ78840	9.3E-07
	Chv1VELVK55_0060004	1363	339	95.56	AEI58657	9.23E-63
**SPCS2**	Chv1VELVK65_0011192	963	193	251.15	KDR10695	5.6E-115
**Protease inhibitor**	Chv1SOAPK55_0010469	414	99	1441.73	XP_005304994	2.32E-09
**Serpin**	Chv1VELVK65_0051330	1791	387	104.68	KDR09404	1.73E-48
	Chv1VELVK65_0051329	1605	325	49.43	KDR09404	1.84E-19
	Chv1VELVK55_0006994	1300	410	67.27	KDR09404	6.16E-69
	Chv1VELVK35_0011937	1165	323	19.7	KDR09404	1.62E-56
	Chv1VELVK75_0000424	1586	407	102.13	KDR19769	6.1E-99

MME: Membrane metallo-endopeptidase; GGT: Gamma-glutamyltranspeptidase; FURIN: Endoprotease FURIN; SPCS2: Signal peptidase complex subunit 2.

In *D. melanogaster*, most seminal fluid proteases are serine proteases, which break down proteins into smaller peptides by cutting the protein serine-protease cleavage sites [94]. Serine proteases are required for the localisation of the sex peptide (SP) to regulate sperm storage parameters in *D. melanogaster*, one of which was inferred to act upstream of an astacin protease to process SFPs involved in ovulation and sperm entry into storage [94, 95]. Trypsin is also a significant factor involved in releasing the mature or activated SPs in the hemolymph of females, and the mature forms of SPs contribute to both short-term and long-term post-mating responses such as inducing egg-laying and inhibition of receptivity [96]. Carboxypeptidases hydrolyse the carboxy-terminal of a peptide bond, and together with serine proteases, they are thought to be important in spermiogenesis and possibly post-coital stimulation of females [92]. The presence of ACE in seminal fluids is also essential as feeding ACE inhibitors to a male *Anopheles stephensi* resulted in a reduced egg-laying rate of its mate [97]. In addition, these proteases are mostly regulated by protease inhibitors that specifically target the recipients to avoid causing damage to normal cells or tissues [98].

We also found other *C. hookeri* transcripts annotated as proteins that have been reported to be essential in spermiogenesis and/or regulating of female reproductive function. Three transcripts were annotated as serine/threonine-protease kinases, which are believed to be functionally involved in sperm capacitation in humans [99] and the recognition of sperm chromatin during spermiogenesis in vertebrates [100]. The abundance of serine/threonine-protease kinases in the testis-vas deferens and male accessory glands but not in the spermatophore of *Dermacentor variabilis* (tick) suggests they are likely to execute a similar function in insects [92]. We also found a transcript annotated as transmembrane emp24 domain-containing protein that might be involved in ovulation. In *D. melanogaster*, a similar protein is present in both the central nervous system and vitellogenic egg chambers and is believed to regulate female ovulation through the sex-determination hierarchy to control female egg-laying behaviour [101]. In addition, transcripts annotated as lipases were also present, whose activity was detected in *Drosophila* male accessory glands and transferred into female bodies during copulation, providing energy to sperms through hydrolysing triglycerides [102].

In summary, male *C. hookeri* produces similar SFPs when compared with other insects. However, there are numerous transcripts lacking Blast hits, indicating the presence of novel proteins. These proteins, together with those SFPs classified above, provide large resource for the study of sexual reproduction, speciation and parthenogenesis in the New Zealand stick insects.

## Conclusion

We report the transcriptome of the stick insect *C. hookeri*. The assembly comprising RNA-Seq reads sequenced from female antennae, head and prothorax, leg, midgut and male terminalia, was assessed to include a broad representation of expressed genes, and provides a comprehensive resource for analysing genes of interest for this species.

In the mining of gene families involved in olfaction, we conclude that *C. hookeri* has a fully developed repertoire of olfactory gene families. The majority of these olfactory genes are over-expressed in the olfactory organ of female antennae. Some are enriched in male terminalia (*OBP9*, *CSP6* and *IR7*) and female midgut (*CSP10*), suggesting their roles in sexual reproduction and digestion. Highly abundant PBPs in female antennae, in contrast to their usual function for male insects to detect mates at long range, may be useful for females to detect pheromones released from other females in order to space themselves out in the environment. In addition, recent gene duplications among three olfactory binding proteins (*OBP4*, *OBP5* and *OBP6*) may underlie key adaptive events during sensory evolution in *C. hookeri*.

Identification of the members belonging to GH9 reveals a total of nine gene duplicates in *C. hookeri*. Comparison with five gene copies detected from the divergent phasmid *T. cristinae*, suggests there has been a dramatic cellulase gene family expansion during the evolution of phasmids. The increased copy number of GH9 likely allows this insect to endogenously degrade cellulose without assistance from symbionts and some of them may be able to break down xylan, xyloglucan, and/or glucomannan as has been observed in several other stick insects [18]. However, some of these duplicates might also have evolved new roles rather than digesting plant cell walls as they were not detected in the midgut. We also identified *C. hookeri* pectinase sequences from the transcriptome, belonging to the GH28 family, the group of enzymes that were recently demonstrated to have transferred from bacteria to stick insects [8]. However, these enzymes are most likely to be endogenic in *C. hookeri*, because the genes encoding them are present in the genome and the two genomic scaffolds containing these genes also harbour other insect genes.

Our identification of accessory gland proteins in *C. hookeri* represents the first molecular genetic investigation of sexual reproduction in the phasmids. The male terminalia containing predominantly accessory glands were enriched with SFPs. The predicted transcripts of protease/peptidase and protease inhibitors that are over-expressed in male terminalia suggest they are essential to sexual reproduction, and most likely regulate processes through proteolytic cascades based on findings from *D. melanogaster* [94]. Many putative SFPs lacking Blast matches indicate at least some of them are either novel proteins or highly diverged.

In conclusion, our *C. hookeri* transcriptome reference, together with identified gene families, provides a comprehensive resource for studying the evolution of olfaction, digestion and sexual reproduction in phasmids. The duplication events in olfactory binding and cellulase genes in *C. hookeri* may underlie key adaptive events in the evolution of the stick insects.

## Supporting Information

S1 Table Table of over-represented transcripts from libraries of antenna, midgut and terminalia. Transcripts were ranked based on their abundance (TPM) in the sample library. Transcripts from the terminalia library were added by signal peptide check and sub-cellular localisation.
(XLSX)

S1 Text Amino acid sequences of predicted *C. hookeri* ORs, IRs, GR and SNMPs. (DOCX)

S2 Text Amino acid sequences of predicted *C. hookeri* SFPs of protease and protease inhibitors. (DOCX)

S3 Text Species and accession numbers used for constructing ORCO and GH9 phylogenies. (DOCX)
