# Supplementary material for: De Novo Transcriptome Analysis of the Common New Zealand Stick Insect Clitarchus hookeri (Phasmatodea) Reveals Genes Involved in Olfaction, Digestion and Sexual Reproduction
Source: PLoS One. 2016 Jun 23;11(6):e0157783. doi: 10.1371/journal.pone.0157783 (PMC4919086; doi:10.1371/journal.pone.0157783)
Supplement: S1 Text — (DOCX) [file pone.0157783.s002.docx]

>ChooOR1_partial Chv1SOAPK35_0009687

EKVRNLLDSFLFSYFVVSSLSMCLIVYNIAMMEAANPRMMMCISYLVFTVAAIGLFCFFGERITQKSAQVNDIAYATDWMLYSQSFKRSLLMIIMRSQKPVYITAGMFGVLSFSTFAGADSTSWLFVFHSAEEPWSRMKPLLRTQDFCSRQKIHAAYYCIKIIIYSH*

>ChooOR2 Chv1VELVK29_0021980

MENFPGSSNRDNPASKQAVSLTLNILHLAGLWPLKKSGIYTMYTSAVSLNYCTFLLMMAWTLIAGGKTTTSSLEDIGISMAFIPVTFNVAMVLFKREQFEKIITNLKTFYARTLNDDAVKSLHDAAAEEKILFNAIPIACVFGLTAKLCQPLMGYYTGHRDVNRTVEINLPFQLHPLEDEASILAFTVMYAFQCVVFSFVFWAFIPADLFVIVLILYAAKQFEILTILIRSVDCRKRRQLLEVGEEHLQVTPACRRELDTKKSGEAKRVFVNTELEDEFSTVGNDRKTNSGECEEMANVWRKMAAYHQEALRNATDLSELLRTSLFLVYAVTSLVICLILYQLRTVDGVLLIVYNGNYLAAILARLFMISFFSTRLASKCVEVSDAIAGTQWYDYGPGVK

TTLVIIMCKAQRPVKMWAGRFAVMSLETYAAILQAAYSYFGMLVKMT*

>ChooOR3_partial Chv1VELVK29_0015825

RNLCELLRTIFFLHYMTASLEIALLLYAINGTEDTSQKVTLYSYFVAIMIRLFIICYFATNVTSKSTEVGDAICTTNWYDYSQEDKQLLLIMICRNQRRVELWAGKCAVMSLETYITILQAAYSYYNIMDALR*

>ChooOR4_partial Chv1VELVK29_0033146

MNSAEEIFDHLYPCCCLISVSTRLFILNLLSTRLTTKSLEVADAVADTQWYNYSSEDKSTLVIIMCRAQRPVYLWAGRFAVMSLETYAAMMQAAYTYYNILSSLS*

>ChooOR5 Chv1VELVK35_0019903

MALPAHFGSTAKQQSSVQAMSLTMKLIRFAGMWPLPLDGIIYKTYSCTVCCNLILFMIFIWLSTFSIWEDLFTLIDNMAFLFSLLPPVFTMVSIFKNRKLYNEVIASIRKLVFYNKFDDENELKKSVRRALAHEKKLLVGFFTMVGITLISLMLKSFIMLLFGEKAAGESEMPFQYFSKDYKAGLVFFTIMYTHQLLSIITIWVSAVPLDILNFQLIKYVTQQFEVLCIKLTNFGANQENMYEGTEDDDSVQKPRCKVVTEHILNLDCDYVNFKTDVYIPKNSNKKTNNNISGKANNKSQEILHKLILDHQEILSNAEKLCSLMSSYNFVHYLVSSGLICLLLFISTVVESKDMLLIKGSHLISVICRLLVSCYLSSQLSHQSLAVSEAAYSCNWRFLDKDCKSALVLIMCRAQRTVTMKASSFAVISLPTFLTLVQCAYSYFNMMTTMQS*

>ChooOR6_partial Chv1VELVK45_0034664

MDYVEYHDMAFRYVKELRNVFSLVVLSQVVMCLGIICQAGFLIVVNKGAITDFLKYSGYLAVTLFELLMYCWYGNEIIYQSSTVQSAAYESPWVNGSPQLRAGVQMAMLRSQTKVALTAGGLHTVSLSTFTAILKNSYSLFALLRQISES*

>ChooOR7_partial Chv1VELVK21_0035864

YYKDHLHANSTVKMGLPIGMYPLGEEENRVLFAALFVFQAMSLTLTFVAFAGVEIFVYTLIVHTANQFEVLKILIRDIESGDWLRESAEGIRLQSLPERKRTGLKEFDVVETCSGDAGVRNEMPMVHIKGKPESEKNEVFSCFWKKFSAFHQEALRNADTLCELLRTLLFLHYALTSLILCSLLYQIIVREEIFERVHMCSYLGVILTRLFILSILPTILTTKSAAVCEAVAASQWYHYSSEVKSTLVIIMGRAQRPVYLWAGRFAIMSLETYAAMLQASFSYYSILKKIA*

>ChooOR8_partial Chv1VELVK21_0058449

NSSITMPSSLIFLTVLTHRGNIRSLCEAVNAFYDQPEVAHHLHLLERTRRIMRNLTIGTLTMGVLDSLMWSLNERSVTSVFVYVNQTNLLPFDRYLPYEPSESTLGYYVAFFLFSFEFTLSVCTFLSTDMTSLALLAYGSTQLEILLRCLAHLRQDSVEHLRTVQSRVVRPTYSQDISKTQKGEYLCSDTLSMEARGQKTFSAEDHGSNIVSKEAHKEAAGINDIPEVEIVAEMKRRLAVLVEVHRSILRYINDIENMIGMLMIVVFFALTFMICLSLFQGVTRDMSFRQTYKFASACIHATIEVFIYCRLGESITSKSDLVGTTLYMTDWYDEPADIKKAYLIIMSRSDRPVIVRAKPFYVVNMATFVSVLNAAYTYFNLMREFNN*

>ChooOR9_partial Chv1VELVK21_0062558

MTGGGNILERAQLPLVTYPWLDRSDPASFVVEYVVQACNASALMVVTLAADLFVIFVIKYSAHQFQTLAVFLRATGRRRLRGPGRRGSGDGKADVGSGSSGEKTDEETLNTLIQYHQSILENAKKLANLLSPTLFTDYVTVSIVICLLLHRIVMTENTAENGTKCTHLASSLTRLFIVCYWSSELTYQSLAVEDAVYACPWYKYSSATKQSLCLMICRAQRPVLVKAGLYAVMSLETFSSLIQGAYSYYNFLKQLSTS*

>ChooOR10_partial Chv1VELVK21_0073095

CLSEQNFDTYDNCSEEVTFNNVDSSTAERQSVALELKKIDGALTILSEWHQVTLTNAEEIVKLLSPVLLAHYLVSSLTICVLAYTFQNVRDKSQLIALTSHLVLVLSRIFIFGWFSSEVFSESLLVADDAYFCEWYDLSYGAKKSLIIIMARAQRPIVMKTGPFGSLSLKTCGKLLEAAYSYLNILNNIDAM*

>ChooOR11 Chv1VELVK21_0077746

MDVLGKEQLMNTEKHIGMSLTTKLLRYGGMWPNINDKYYKAYTCIVALNCVLFMTLLSHIYFSDGKDLSAVIENTDHILTVASYIVTMLSILNNRGVYKGIVSSIQSLISYSQSDVDDDRKRAVRKGIHIERKLCKYYLTTISMTASCFLLKPLVTIVMSSHEINLASMDFPLKLVPFTDRTSILKVVFEYLCHCSILGLLIITIVPLDLFTFTLIKYLAKQFEVVSILLNSVGDHKHDRDNVSARSLESSDSLNQKSKYNIGHFGSLNEHHGEDFCSGDVIRGFKIQAGHEICRNRIRNQMRAENDLKMLVKCHQEILENAKKLVRLLSPIIFSHYLVSSLTICLVGYQITTSVSVCHAAYSIGWYPLSIDAKRTLSLVMLRAQRPVCMRASQFGVVSLEMFSALVQTAYSYYSVLIKMNST*

>ChooOR12_partial Chv1VELVK21_0088043

MPVGFQFDTENLAIYLLLFFYLSVAVSSLNLAFGTIESLCIASILHLNAQFRILHNSLVNLKQDAEAEIKNNNNGTRVLEADVDEHFLPTRRRIYEQPATDILGNRRMETGRSETSYQSTEKFKKDFCKEMEHRLIDCIKHHNNILKSAGKLTSVYSSMMVVMVLVDVANLTFVPFGAMTSFQTGAGLSVLWRYLNLFMCALFHSCFYCYTSEQMASASEMVATAAYNCDWLEAPLSVRRSLVMVIARAQRPVSLSIGKFTRLTLSTLEWTLRVSYTYFALLQQVYNRGE*

>ChooOR13_partial Chv1VELVK25_0038317

CGLYYIFYDAVFVALSLHVCCQFDILKVSLRAIAQGNCFSAQPRAGPAGEDGVTTDRSVSAVSKHEKPETARFSKLLQCIKHHQQILSVFAELNELLSPMIFIMVVDNLFKMTIVTVDMVLTPVSDINFGGILQLQICTMIHAILYCWGSAEITWQSEMVLDAAYECGWTEASGHFKQLLRVLMIRTSRPASFTIGGYGPITLQTFLGMTNFAYSVYAVLRQTYN*

>ChooOR14_partial Chv1VELVK35_0042637

CMSASSFDALCVAFFMQVAGQFRILQTSLRRLQEVAEESLARSVTYSEAEDGNAVGPSSSIPDGSTDCRQEALKKHALQHLKYSIKHHSDIIMFVKKLDDVLNPIMLAELLYSMSEIAISGLQATESSADFGNLLKFLVFAVSLSFELYFFCRYADVLLDLSQDIAITAYSSEWYKMDPQIKTMLQIVIMRAQKPVKVTAFHFKVMSMDTYGALLNGAYSYFALLKEIKKGK*

>ChooOR15_partial Chv1VELVK25_0067528

NPGSVIYSNVQTLADLLSPLLFTHYIMSSANVCLIGYEAAVAEDTTLFLAYVGHLVTVLSRVFLLSYFSSEVMHKSMDVSQMAYFCNWFPFSGNFKRTLAMVMRRAQRPVVMRTGGFGILSLETFAGLMQAAYTYYTLLKKFNIS*

>ChooOr83b Chv1VELVK21_0029367

MQKMKVTGLVADMWTHIRVLQIAGHWLLDINKSSSAMWNYMRVAGTSLQTFLLVMNYVFMIINCTQQTADSDEFTSNVATILFFTHCIFKLFYCAMRRRKFYRTLNSWNNSNSHPLFAESSARHHANATSGMKRLLVIILTCTLTSALAWTTVTFFGESIIHVHDPENENNTLIEEVPRLMIRAWYPWDTSNGFMYGVTFLYQLFWLVSMLVLCNLLDTLFCSWLIYACEQLVHLKEIMKPLMEISHSVDSLMPQTADLFRAASSSSHAALLSGSADALDANVRGIYNSASEYSGFRQGVGTLATIQSSSVGPNGLTKKQELVIRSAIKYWVERHKQVVRYVSDIGDSYGGALLAHMLISTVELTLLAYLATTITGFNPRGICIIGYVIYSFGQVFQFCYYGNHLIEESSSVLEAAYSCRWYDGTDEAKAFVQIICQQCQKSMTISGAKFFTVSLDLFASILGAVVTYFLLLIQLN*

>ChooIR1_partial Chv1SOAPK25_0135053

VLRTHELYSGREDASRVLTSWRRDGLARDVLLFPDKLCRGFNGHRFVVAVSDQPHYVIHRNTSGNNTSNYTWEGLEIRLLRLISQTLNFKIQFTNFPATDRRSGQASGVVAELMAHRAHIGLAGFYTTTAILKRLTLSAGHSIDCATFITKTSTALPRYRAIMGPFHWSVWLALTLTY

>ChooIR2_partial Chv1VELVK21_0063588

FNTTVWLCSAALLVLCGALLLAALRWEKMATDNNYRLAMGVSQKVSWTDIVLLSVGALCQQGSPVESKGTPGRIISLQLFIFVMFVYTSYSACIVALLQSSTNTIQTLKDLLDSRMKLAVHDIVYNRYFFERAEEPVRKAIYQRKIAPPGGPPRYIELADGVGRLRKGMFAFHFEVGTGYKLVLDTFEEDEKCGLTRIPYIQVVDAYQIIQKGSVYKEIMAIAYNKLIERGFQKRNWNRYYTGKPECNSRGSSFMSVGLIDCYPVLVVLLLGLLTSVLMLLLEVLAHRRQEVHQRMFSAVSKTTFGVT*

>ChooIR3_partial Chv1VELVK25_0038786

TPGRRFLVSANMMLPLISIYSLAIKLVSAQKIFKILVLTEENQAPVLEMMAASLKAAEEKYLGVQFDQVPVAVDRENEDESFEQVCKELTGGVSAVLDVTWTGWRKAQTTCNSAALPYFRADVTPGTFVDAVDAYLGGRQATDAALIFQNEQELDQTLYYMIGRSIIRVIVLGGLEGNATERLLAMRPSPSYFVVYASTGNMTKL

>ChooIR4_partial Chv1VELVK21_0041817

MASHLDKRGLEIAPRPVENCADHAHNTSPPENKFYGNATQVMKEMGEDRVIGFAPADSLLYLRGDVDVWMVNASEKQRQGSFSREKQLALAPNVTLHSGRRFFRIGTSESVPWSYKLRDEVTGELTRDEKGRPVWDGYCIELLKDLAATLQFDYEIVPPKDGSFGSRSVDGSWSGMVGDLATGETDMIIAPLTMTSEREEVIDFVAPYFDQSGISIVIRKPVRKTSLFKFMTVLRLEVWLSIVGALTVTGIMIWFLDKYSPYSAQNNKAMYPYPCREFTLKESFWFALTSFTPQGGGEAPKALSARTLVAAYWLFVVLMLATFTANLAAFLTVERMKSPVASLEQLAKQSRINYTVVKDSDTHEYFKNMKNAEDVLYNVWKDITLNSTSDQSKYRVWDYPIKEQYGHILQAITQAEPVQNATVGFQKVIAEEDGKFAFIHDAAQIRYEVSRNCNLTEVGEMFAEQPYAIAVQQGSHLQLEISRRILDLQKDRYFETLSGKYWNSSVKGTCPDTDDSEGITLESLGGVFIATLFGLALAMVTLAGEIFYYKRKKNNTVSIQKPGANSNALSAKKQITIGKEFRPVVGDKGMPRVSYISVFPRNQLY*

>ChooIR5 Chv1VELVK21_0061997

MGPRCWMLIAFIFLLDHICALPQDIRIVGIFHKDVDDLQDAAFTCAIERLNEEQKLLPGSRLVPEVLYVLSHDSFATGKQVCNRTQSGIAAVFGPQSPSSKGIVSSICDMMKLPNVQTNWDMNVNTETLSTLNLHPHPDLLAQALVDVIKYLEWKSFAIIYQTHEGLMSLQEVFKSRDKSLTTTVYQLSEDGDYRAMFKTLSKTSQTHMLIDCDTDLILEVLKQARDVNMLGDYENYLFTSLDSHTLDYIEFQPSMTNITFLRLVSPSSPEMQEAKRDWDSRGCRHVPPDNMKVAPALMYDAVSMFAISLIRADASQPIETESLSCARPREWSHGEHVNTYMKQFKFLGLSGEVSFDVLTRRRKDVTLELMEVSHAKLRKIGNWSMNIGITEEKDYGEQVSQEAKAMMRNKTFIIASKIGPPYLDWKNRSATGNDRFEGFSLDLIDAIAKFCKFKAYEFVIVTDNQHGKLIPETGQWNGIMGEVINRRADMGICDLTITFLRGSAVDFSAPFMNLGISILLTKPTKDPPELFSFFFPFSFDVWVYMATAFLGVSLILFVLCRITPHEWDNPNPMEEDPEELENSFNLLNCLWFTIGSLMAQGCDLLPRAVSTRLLAGMWWFFTLIISSSYTANLAAFLTNSRMD

>ChooIR6_partial Chv1VELVK21_0029672

MAGMWWFFTLIISSSYTANLAAFLTNSRMDDTIESAEDLAGQTSVKFGSLRGGATAQFFEASNYTVYQRISAMMKQTKPDVFTSTNQEGVDRVLKEHGKYAFFMESTSIEYEIERHCELTQINGLLDSKGYGIALPINSPYRTFVSEAVLKLSENGKIKDIKDKWWLVKNGTGCSEIKVEEENADELTMANVGGVFLVLMVGCFAAFLVAILEMLWNCRKIAVEEKITPLEALVSELKFAVNLSKTTKPNRKKKSEKSSISVCSSVSSSEHVQDSQSNRV*

>ChooIR7_partial Chv1VELVK25_0009676

MLNRGVPTDNYIISEDKLIRLSRLDQRMNDDYNFTSFEESVGFPAHDSNMKSFKYIQELCNFQTILNITGELAGLSTTDNEGMIGMLSKQGFDVAATFSGVLPLTFQYVDFLQPTTRFQPMIVFLQPTVPSLRNVFLLPFSHTMWLVLLATTVFSTTVLLASVKLAEDPSQPWSVSDVILLMAGIICQQSVFRLPRSVSARTTIAVLTMFTILIWSTYSGEVMSFFSTPDKPIKSILHLVQFKYKMGIDAYIYDSMFLLEDAVLQDLKADAQVLDTNDGILSLRKGQYCFCSAALLVEETSKNLLSTSELCQMTFFSQNNNIQLAMTVPKKSQFKEALNWGVMKLRETGINEYLLRFETIDLPECANDVSDLDMTELGSAFAMLGMAYIVSVIILVAELTLNYTSTNDLTISSLTKDHHGLPKRTRQQSFPHKNPVPAVDLWNLP*

>ChooIR8 Chv1VELVK65_0046897

MDPLTLPHCKLEPLVVNVSTSNSFAAGHRVCELLQRGVVAVLGPHSLMSAGIVRSTCDSKHVPNIQTNNPEVLPSRHPSSFYLNLHPPPHTMGKAFVEVIKYLGWKSYALIYETNEGLMRLQELFKSRSATDEIIVVRHMRKENDQRPLLKQLHEASHTHFVLDCDADRVVDVLRQARELGLMGDYESYLLTSLDAHTLDLSEFQYSQTNITALRLVSADSTAVRGAVQEWTTGEEMQGRHLDMRPQTLRTEAALMHDAVRLVASALHQINIRSPNYTQPVSCDSGQTWRHGLGLANFIKMVKVKGLTGMVSLDENGQRVDFTLDVMEVYHGGMRPVGTWSSKTGLNHTQAFTEKLSEIQRNIQNKTFIVSTKTGMPYLGWKEPRGGRVGNDRFEGFSVDLVQEIASQLQFKGFELRIAEGNVHGTLDQETGRWKNGIMKDIVERRADLGICDFTITYQRGQAVDFTVPFLTTGISILYTKPKKPEPELFSFLKPFSFDVWIYMATVFLGVSVFLYVFARMTPNEWKCPHPCNREPQSLENSFNIANCLWFSIGSLMAQGCDILPKAMSTRLLAGMWWFFTLIMISSYTANLAAFLTITRMESSIKGVEDLAYQSKVKYGVLRGGSTSSFFQNSNHSLYQRMWAMMSQAKPDVFTATSLEGVERVQRSKGHYAFFMESASIEYETEYRCDLMQIGGLLDQKGYGIALPIDSPYRTLLNHAILKMAENGVLADLKTKWWRTRGGGKCDSGKEETAGADTELTMANVGGVFLVLLSGCLVAFLLAIIEFLWNCHKIAVRKKITPCEAMVGELKFVMNCSQTTKHLEKNHENDTVSQRSS*

>ChooIR9_partial Chv1VELVK21_0053197

LLVIAVFSPALAILQEDVLQLVDDYFDYIRVRHVTLFTCSVLDSLRLARRLGAARRSWVFAPGAGQRAATLDTAARRDYHKYGVFLDHSCAAGEQTLQLNSARQFLNGSYHWLVWSAAGGDSRLEVAQQLNLSVDSELAWASSSAGGDGGVELHDVYRVGPGMPPMHTLAGLWTRDQGVRYLLTAYKYLRRTDLAGLRLRTGITINTPLENPEVNLLKLENRQLDAMATYNYGLYILLQQQLNFTMELVVTDKFGFVVEDGNYDGLTQLLGERQVDTAISTLLMNRPRMSYVDYSTGYGWEFHICAIFRHPSVSGGQDALIKPFSGSTWLCTFLMWFVIAAFLKLLAWVQPLYADVTGEEEEEIPSWSDLMLLVVGIVGEQGTWLDSKWITWRFVFFMMLVLTVLLNTYYAAIVVSTLLNQPAQTINTAKDLIDSHLHFGAEDIIYNRPYFEVNSDHLVQDLYKKKMAGHNSYFPMEEGVKKVLQEEFAFHTEAVRAYPVIESMFPDEKKCALKEIEFFPVEMGFFAFPFMSPYKKMFTYGLRKIAQTGLWQHQNHEWRS

SKPSCVSASAEVISVALPSLTPAFALLAIGYVLSVAVLALENLRHTRRRRALHVSPF*

>ChooIR10_partial Chv1VELVK21_0109764

AISLDSKWVTWRVVFFVLLMVTLLIDTYYSASIVFSLITEPPKTIRTVKDLINSELEFAVENLSYNIPFFQMNDDPLVHELYVKKMLGARRTQPVNYSRQEGIRKVLNEKFAFHSEPANVYPIIAATFPDPKKCSLTEIQVIPIEICYMPVRWKSPYKEIITYGLRKVAETGLLYRLNSIWRPPKPRCFSGGGDFVPVAIHTVVPAFAILLCGAIGSTTVLLLEQHWFKRSKK*

>ChooIR11_partial Chv1VELVK25_0018305

TNKPAQKAGEDFPDISAITVPWLGIADSVAQKKAFLGAAALTMTDRSKLLLNFTTHIGIQPYSFLVSRPREVGRAFLFMYPFNTKVWLCIIVCVATMGPVLMLFHQKSPYYDYHGKRGKGGLNSLYSCIWYVYGALLQQGGRHLPEADSGRIVVGTWWLVVLVMVTTYCGNLVAFLTFPKYENSVTTLEELLEHPAGISWGLLGGSDLANRLQSSDDARLRELFDGAMKHPDQGLKVVQEVRDKKHVYLEWKLNLVYLMKRQYTATNHCDFVLAKEDFWEEKLAMAISANNPYLPIINEQIKRMHKTGLINKWLEQYLPPKDRCATVTQQAAEVTTHVVNLDDMQGSFFLLMLGVVGSLFLAAGELCFHCKKLAAEKRLIRPYSP*

>ChooIR12_partial Chv1VELVK25_0043548

SGGTDALVRPFSGGLWLGMFFLWFLIASFLRLMAWLQPRYADVTGESHEDIPSWSDLFLLVVGIVGEQGTWLDSRWMTWRFVLLMMLMLTVLLNTYYAANVVSTLLNQAPLTIRTLKDLVDSSLQFAAEDVIYSRYYFEITNNSLIQKLYAKKMAPKGGNYISKEEGLQRVLMQEFAFHMEDVGAYWTIEHTFPDSKKCALQEIELFPGEMGYYVYPFKSQYKKMFTYGLRRVVESGLWDHENRRSRPQRPTCSSKGAEVVSVDLISLTPAFGLVVVGCTVSMLVLVAELIEARIAGQ*

>ChooIR13 Chv1VELVK55_0002278

MGYHLRKRVMFIVIASVCDVLNNVRGHVFRDALLGLTDRVSKNTSAECIHVFDNVEPHSDIFIETQLVHCQKLLTNCRLPTMFSSVNTTINMKALSTRCCKPLYVIVVSDTFETRNVLRKIMEEWCHTKITWLLVPLSHYPMHQFYTEIHNMGFNCNKLVVTSTRTGPNSQLQALDYTRMAARNGSQNWKQHCYERKTDLNGVTLRAVTRNQPPLITIQKVGDRTESSGFVGELWKLLEETLNFRTELITDGDVSPGITLKNGTCTGMAGAVQEGRADIALQLIKVTAPRLRCGQWLTPWETVSLRIFINMPVDGSSNTQRYASAFACSVWLSVVVCVLLLGCSLAATFNTSPTTTEGWRRTFGGLAVLSESVLATAAAIICQQGYHRKPRTYSACIVILLCYMTGVVLMTSYSGRATSHSAIKKIKLPFTSLQQMNADGTYQLSVIANSSSHNMFRTSKSGFLKNVYEKQMDHDRRNYPKSLVEGIKNVCSDEKYTFLGPYELMRPVLKRANCNVIALPEPFSKGPFGLLFRKNSPHLRPINSGYLKVWERGLRSCLMLRFLRTTVHSNSPKHRIVTLKDVEPLLLTCTLGCLLALFILAVEVTLPFVLKLR*

>ChooIR14 Chv1VELVK21_0047941

MSAGECLDTDLLLFLRDVLGGTVVVASCWPTRQSMELVKALSQSEIMTSMQDCSGRISVPVPCQPTTFLLDLQSKNAARLTRQADEMDLFSSPNRWLMIMAPGVITTSNGTESEVPDTSSPADYGPLKELLSLLSSMKVFVDSQVTVARRLEDGSFSLMEVYRMGPGQRAVVRTTGLWQRQSGLRTTELSLPARRTNLHKTRLKTAMVVTTNDTLDHLTDLHNKHIDTITKLNYILLLHVIDIMNASIEFTVVDTWGYPTNGSWSGMVGYLQRKEADIGATALFFTANRLPVIDYIAMTTPTRSKFVFRQPPLSFVTNVFTLPFSQGVWLSSAGLVGVCTVVLYCALSREAGQSPSRTGVAWSDVLLLSLGAVCQQGSQIQSSEPSGRIVTLFMFVAVLFLYTSYSGCIVALLQSSTDSIRTLEDLLNSRLEVGAHDIVYNRYYFETATDPVRKGLYGKVLSSGSKTPNFLELEEGVEKVRRGFFAFHMEESSGYKVVLETYTEEEKCGMHGIGGYIQVPDPWVAVQKNTPYKELMKRGYHRVQEHGLQSRELSRLYTRKPECSGHSSSFVSVGLVDLYPALLVILAGTIISLLSLLLEMVVHKKCCASSVSLLEPPRRLSALPAHSHSGRSTTEVID*

>ChooIR15 Chv1VELVK21_0052279

MLFIRDYFSRRDISILVAFMCSNADIHKLMKFLSLTSKMWVFAPGIEDRTATLSTIALQTFYRTGVYLDYTCNSGREVLFLNSEREYLNGSHHWLVSSDSPQVPREVDELRLRLDSELVWATRENGTITLHDLYKIDHNWPVVRTVAGSWHVSTGLRYYLTQWKYHGSRRGDLQGLNLLAVLIVTDIPPENIDEVILEPESKDKDTMAVYNYRANTHLRRLFNFTVSVVTTSEYGYVVQEGKYEGMLQLIGDREANISISSMIMNSQIRLDYVHFIAGPTWKFRLAAIFRHPPVTGGYGALLQPFSPELWMCAMLMWLLIAVLLRLMSWVQTAASSTAHDRRHDDIDDKHSWSDIMLLIAGTLGEQGTDLNSRWVTWRLVFLTMLVLTVLLNNYYGACVVSSLLSEAPLNIKTVKDLIGSSLTFGAEDNNYNSKFFEFDSDPLVYELYQKKMLGPRGRKPVYYSREEGVRKMLHEEFAYHTEVITVYPLIETTFPDKEKCSLVEITVYPTVKSYIIVPFKSQFREILTIGMLRLGEAGILTHMNLELLPQKPTCSSTGDLISVGVVYVVPAFVILFVGVIMSVLVVILERACQLKNQ*

>ChooIR16_partial Chv1VELVK21_0091436

VQDNDDEEEVVDPGCILRNPPLLTHIHLKIATITDHPLSYVVTENGKKVGKGVVFEFVEILRSKFGFTYDVVTPEENVIGNNKTGLLGMLHRGEADMAAYFLPLIWEKSHGVRYSFSLGDVDWVIMMKRPTESANGSGLFAPFDTTVWLLILVSLILTGPVIYLIILVRVKLCKGSERLAKIYPLDACIWFVYGALMKQGSTLSPMTDSSRLLFATWWIFITILTSFYTANLTAFLTLSRFTLPIDDAKDMADYHYKWMAQKGLTMEEVVRFDPTYYYLKDSYKAKRGFFMAGDSAKMMATVQKENRMFLRERNVVEYLIMRDYNDKTHRGVDEVKRCTFVSTPKAFMERSIAFAYNPNSTLYKLFDPVFMGLVEQGIVKHLLRRGLPKNEVCPLNLGSKERQLRNSDLFMTYIIVMSGYAIAAIVFGSELVLRVMKKFGDSRVISTHEAGYVPSKSHMFPPPYSTVLMGLDPEGGGKKQSINGRDYLVVNAKDGESRLIPMRTPSAFLFQYSA*

>ChooIR17_partial Chv1VELVK45_0004605

FSMTPLRIDVVDFTFPLIYTKNCVFIKHVNTDSSTVHWTAYLAPFGVDMWATIGVFVLFMSAVFFVAYNLTHKYVVGDEPPERSSYIDCLVFVFGAFCQQGLDVNTRTCSLLTLSLATYLTAITVYASYSAQLISYLTVREYQLPFETLEQLLEMGTYRLGVLANSGQLNNFNKATDPLMKQLYASLIAPDKDDLPPTIEEGLRRICEIDHYAFMTSFDVVLGLLENVSCNVVAVPFASYKESLAIAIAKNNSYKNLIDNTLQVLRRNGVWHKLRVTEWPVHMPVAEESLESVSLVRVMPLASILATGITTALLLFCCEYRHRIRARIASWRLP*

>ChooGR_partial Chv1TRINITY_0207975

ALRGDPSSRLAPVASPELLHGEGRRATSTRRGARVHRPPRDDPAEQLKTFSMQLLHDRFDFHACGFFPLDYTTLCSMVGACTTYLVILIQFQLSNEQCAGNRTINES*

>ChooSNMP Chv1VELVK55_0003575

MKDAAGIKAWFTKTPCVAAFGVGGALFILLGSCLGWAYLPGLLDRKVVEKVVLVDDSPAFERWRILPQPLVTKFYLFNVTNPDEVQNGARPVLNELGPYVYDEHIERVDIVKNQDGETVSYNLKSTFFFNQELSGNKSTSDSVTIINLPLLGTALKVKKYFPFALKLMEPILGEIFPYSGSIFLTGTVAQLLFEGMTVVDCSNTSTEIAEMVCDQLETQMPETVERTDENTYKFSFYHYKNGTSKARYTVDRGVTDSKRLARVVAYDGKDRTDAWQESRCNLVNGTDGIFFSPLRNSDDPLVVFSSDVCRSVTLKFERKSQFRGLPVLRYVNDPALLGDPEEYPDNKCYCVGRSNARTCLKGGVLDLSPCIGVAVILSYPHLYMADPEYLQYAEGITPDRRKHQTFLEVEPRTGIPLQGSRKVQLNMFLTRIPEVSVLSNVSEGLFPAMWMDEGIEVNDKNLVLLHSLNNMVNTFYTTRWMAIILGLALCVAACMGLYSRKKNCGSYDNRIIPFEKQGALNMAFVNDDGSKPTPPPDVTKASPPFHAVVDNRRGSAAGDTTKKESSTMTTEPKRVESSCSTLDPSKKDPEGEVKSRSPTGPEDEAQHGSPNPPETKDVTTMGSHRNSAVSENVASPPEDIATSRHPSADSNSSVSSAEDTKSTTSLRSVEEAKGPGSNVVSAKCTDGEDAR*
