# Supplementary material for: De Novo Transcriptome Analysis of the Common New Zealand Stick Insect Clitarchus hookeri (Phasmatodea) Reveals Genes Involved in Olfaction, Digestion and Sexual Reproduction
Source: PLoS One. 2016 Jun 23;11(6):e0157783. doi: 10.1371/journal.pone.0157783 (PMC4919086; doi:10.1371/journal.pone.0157783)
Supplement: S2 Text — (DOCX) [file pone.0157783.s003.docx]

**Serine protease:**

>Chv1VELVK55_0057832_translation Description: PREDICTED: trypsin-1-like isoform X1 [Apis mellifera]

MLPNALLVMFAISCASTEVLFVNRRTPRIIGGTAASIIQFPCVVSLQVELKGNVRVHICGGSIISQVWVLTAAHCGVGRRPTTIKIRAGSTNEESGGWLNNITHIIRHPGFNRGAILNNDIALWRVSPGFVYNQRVLPAPLVSSDDDEVRPGTYGIVTGWGTVNNCSAGSSTLMQVAVPIMSNTECTTFYNPKRISKGMMCAGFREGEKDSCSGDSGGPMFAGGELVGIVSWGFGCARPNSPGVYTRVAYYRDWIAWMTGV*

>Chv1VELVK35_0071739_translation Description: PREDICTED: trypsin-1-like isoform X1 [Apis mellifera]

MRRLHNLASVGSYSSSLRGIGRRPTTIKIRAGSTNEESGGWLNNITHIIRHPGFNRGAILNNDIALWRVSPGFVYNQRVLPAPLARAGYEVPSGTYATVTGWGAMNPRNSGSPTLMQVKVPIMSNSECMKLYNPSRITDGMMCAGFRAGEKDACKGDSGGPLFAGGELVGIVSWGDGCARPNSPGVYTRIAYYRNWITWITGV*

>Chv1VELVK45_0064057_translation AGAP008293-PA [Anopheles gambiae str. PEST], trypsin

MLPNALLVMFAISCASTEVLFVNRRTPRIIGGTAASTSYSSLRVSLQVELKGNVRVHICGGSIISQVWVLTAAHCGVGRRPTTIKIRAGSTNEESGGWLNNITHIIRHPGFNRGAILNNDIALWRVSPGFVYNQRVLPAPLARADYVVPYGTYATVTGWGSMKSRNSGSSTLMQVKVPIMSNCECMKLYNPSRITDGMMCAGFRAGEKDACKGDSGGPLFAGGELVGIVSWGDGCARPNSPGVYTRIAYYRNWITWITGV*

>Chv1VELVK29_0052877_translation Description: ejaculate serine protease [Allonemobius fasciatus]

MLLSAFGFSLIFVHQAWANSDSPIKVNVDTSCDCGALAMVDTRIVGGSLAPPHLYPWMVAILLYGNLHCGGSVINNQYILTAGHCLKWYMIEDLTVVLGTDDRVHMEEGSATIALVEQLIVHEAFASDYIHDTNDIGLIKLKKPLTYSPTVKPICLPGSDDDLRNKTGMVTGWGRTAQNGSPSRYLRRASVKIVDPARCKNSTIGDHIQDTMLCAYEFNTDACQGDSGGPLGYEAKPGKMEQIGIVSWGIGCARPGIPGIYTRVSEYLHWIKTNTAEAVYCQKRF*

**Serpin:**

>Chv1VELVK65_0051330_translation Description: Serpin I2 [Zootermopsis nevadensis]

MFTELVVFPLLAAVARGAVDADMLQEAITTFSFDLLKELVSGEARNLAFSPVGVAAALSALARGAGGNSARQLLTALHLDVSRAAEDPYGGLAKASQEAVSGSAAAVEVAGRMFLDERFQLAAGAPVEVASFGARDGGRAVNDWASRATRARVGELVDARLPADTGLVMASAAFFRSPWSKPFKTRATAVLPFATKPGRVAKTPFMQQSKYLVTGQDDRLAARWLQLPFKGEKFFMLVLLPNEKYGLREMVRKLNYSDISAMTQTYPPKLVNVQLPRFTLTSKIKLDQPLRKLGVTDVFGAEADLRAISSSPAPLWVTLAVHKAVIEVDEQGGLAAGTAGIEVKPRRTNGSETASFQANHPFLAVVFDKSSRVPFLIATVANPSHMT*

>Chv1VELVK65_0051329_translation Description: Serpin I2 [Zootermopsis nevadensis]

MFTELVVFTLLAAVARGAVDADMLREAITTFSFDLLKELVSGEARNLAFSPVGVAAALSALARGAGGNSARQLLTALHLDVSRAAEDPYGGLAKASQEAVSGSAAAVEVAGRMFLDERFQLAAGAPVEVASFGARDGGRAVNDWASRATRARVGELVDARLPADTGLVMASAAFFRSPWSKPFKTRATAVLPFATKPGRVAKTPFMQQSKYLVTGQDDRLAARWLQLPFKLGVTDVFGAEADLRAISSSPAPLWVTLAVHKAVIEVDEQGGLAAGTAGIEVKPRRTNGSETASFQANHPFLAVVFDKSSRVPFLIATVANPSHMT*

>Chv1VELVK55_0006994_translation Description: Serpin I2 [Zootermopsis nevadensis]

MRAGTAVVLLAVALCHSALGKVKKGKKGGDRLQEAVNEFSVDLLKDLFSGEPRNLVVSPVSASMLLAMIDQGAKGHSARQIESAIHLSRRKARLLYGQLSRSLQEQQNSSAKLEAANHVFVARKFKVRPKFSKLLKDTFQSGIERTTFGKGDGANAINGWVRNATHGAIENIVDPVLSSEIAMVIINAMYFQSPWLKPFKTSSTSDLQFNLLGGSTVTVPFMSKRDVFNVGELSHVHASWIELPFLGEQYSVVVVIPNEVGGLPMLVQNISAADIANMISRGDQEEVHLNLPRFSLSTKTDLIPPLKMLGIKNIFDEDLANLAGVSKKKLMVSKAVQRAEMKIDEAGGLAAAATGFELEPRSFVSSLSADRLSPLTSQHIQCCDSKKIYYEILISISQISHKLSSMIFVT*

>Chv1VELVK35_0011937_translation Description: Serpin I2 [Zootermopsis nevadensis]

MRAGTAVVLLAVALCHSALGKVKKGKKGGDRLQEAVNEFSVDLLKDLFSGEPRNLVVSPVSASMLLAMIDQGAKGHSARQIESAIHLSRRKARLLYGQLSRSLQEQQNSSAKLEAANHVFVARKFKVRPKFSKLLKDTFQSGIERTTFGKGDGANAINGWVRNATHGAIENIVDPVLSSEIAMVIINAMYFQSPWLKPFKTSSTSDLQFNLLGGSTVTVPFMSKRDVFNVGELSHVHASWIELPFLGEQYSVVVVIPNEVGGLPMLVQNISAADIANMISRGDQEEVHLNLPRFSLSTKTDLIPPLKMVSPQAGTLLPQHKPG*

>Chv1VELVK75_0000424_translation Description: Leukocyte elastase inhibitor C [Zootermopsis nevadensis]

MMSRSPPAVVGICILVVMLSMSNSAQDVALNPKALDEVSKGSSDFAVSLYQTLRTKESGNLVVSPLSLQTILSLVYHGAAGRTADELATALKFPEDRSLVPSGFYTLLSSLKSDENITLEVANKVFSQAGFGIKSEFNDIARRYFLSDAQEMNFSESTAAAQTINQWVEERTRNKIKDLISPDMLSALTRLVLVNAVYFKGNWRSKFDPEHTTTEPFYTTPTQKTDVQMMHIKKKFRYADIPQLDAQALELPYEGDKISMLILLPHKKDGLAEMETKLSEISLTEIINNMYSTEVNVSLPRFKIEKSIDLNPILKELGIVDMFENTANFSGISAKDDEPLKVSQVVQKAFIEVNEEGSEAAAATGVMFVAFSLPPPPRMFKANHPFIFALWHRTATIPFFLGVHAQP*

**Aminopeptidase:**

>Chv1VELVK21_0040553_translation Description: Putative aminopeptidase W07G4.4 [Zootermopsis nevadensis]

MAARTALAAALWACCVLPVYCSRTRYPPGLNDTQLFSVQVVEELDLRSGSYDAVVFVTSTRLREGKMAPIVRVLEDERKLDAAWDRQVALLEMQEVPGGRLVHSPPGKLVRDYHDVRSIAEATAAGIKRALAAGSKTPLLVLPDHEWFPRCTVASVLAALKELYLPLQLRHDVPSSQGKVDYLGVWTPDRNQFEDVRLAEVLEIGRSVVYDIAASDPEYMTPISIQRYVKNLFANTGIGLQVVSDPDVLQRDYPLFAVVNRGSKAITRYQGRILYLTYEPRGPVRKTLFLAGKGVTYDTGGTDLKTSGSMVGMSRDKAGAAAVAGLMKVLSILKPKGIKVVGAMPLVRNSIGQESYVADEIITARSKVRIRVGNTDGEGRLILADVLCHMKEMALNAVNPHLVSVATLTGHAISTVGPGYTIAMDNGPAREETTAQNLAERGEQLGDMFEISTIRREDYLSHRGKGQGIDVMQQAVQVGRHSRGHQSPAAFLIMASGLDKHGMDDKKPLKYTHLDISPSAGDPPDDGTGCPLLGLYTIFIEQQLYETM*

>Chv1VELVK25_0028117_translation Description: Putative aminopeptidase W07G4.4 [Zootermopsis nevadensis]

MAARTALAAALWACCVLPVYCSRTRYPPGLNDTQLFSVQVVEELDLRSGSYDAVVFVTSTRLREGKMAPIVRVLEDERKLDAAWDRQVALLEMQEVPGGRLVHSPPGKLVRDYHDVRSIAEATAAGIKRALAAGSKTPLLVLPDHEWFPRCTVASVLAALKELYLPLQLRHDVPSSQGKVDYLGVWTPDRNQFEDVRLAEVLEIGRSVVYDIAASDPEYMTPISIQRYVKNLFANTGIGLQVVSDPDVLQRDYPLFAVVNRGSKAITRYQGRILYLTYEPRGPVRKTLFLAGKGVTYDTGGTDLKTSGSMVGMSRDKAGAAAVAGLMKVCWFSQLLSLNPSYALVPCHATEVEDWVRFL*

>Chv1VELVK25_0028115_translation Description: Putative aminopeptidase W07G4.4 [Zootermopsis nevadensis]

MAARTALAAALWACCVLPVYCSRTRYPPGLNDTQLFSVQVVEELDLRSGSYDAVVFVTSTRLREGKMAPIVRVLEDERKLDAAWDRQVALLEMQEVPGGRLVHSPPGKLVRDYHDVRSIAEATAAGIKRALAAGSKTPLLVLPDHEWFPRCTVASVLAALKELYLPLQLRHDVPSSQGKVDYLGVWTPDRNQFEDVRLAEVLEIGRSVVYDIAASDPEYMTPISIQRYVKNLFANTGIGLQVVSDPDVLQRDYPLFAVVNRGSKAITRYQGRILYLTYEPRGPVRKTLFLAGKGVTYDTGGTDLKTSGSMVGMSRDKAGAAAVAGLMKVLSILKPKGIKVVGAMPLVRNSIGQESYVADEIITARSKVRIRVGNTDGEGRLILADVLCHMKEMALNAVNPHLVSVATLTGHAISTVGPGYTIAMDNGPAREETTAQNLAERGEQLGDMFEISTIRREDYLSHRGKGQGIDVMQQAVQVGRHSRGHQSPAAFLIMASGLDKPVVCSEERRQLGEW*

**Protease inhibitor:**

>Chv1SOAPK55_0010469_translation Description: PREDICTED: protease inhibitor B1-like [Chrysemys picta bellii]

MQSAFTMSATRCCLLLLVVCAAAVVTLPKGCCWLPSETGPCFAYMPRYFYNATTQQCEGFMYGGCGGNCNRFRTIENCKEQCNFNLHPRALALMYSCLT*

**Membrane metallo-endopeptidase:**

>Chv1VELVK55_0007423_translation Description: Membrane metallo-endopeptidase-like 1 [Zootermopsis nevadensis]

MKETVIKNPRWWHRRTIMERYLTVVAGASMALLVTVAVVLAILVAQQQQADSLPPMTAAVAGNSNNNNDNKNNVTLACRSSQKQSDVCLTPACVHAASDVLQSIDFDVHPCDDFYEFACGRYVKEKLIPDEKSSMNKFIVINDRLQEQLRLIIEEPIKGDEIKPFQLVKQLYKACMNKSLIEELGLEPMQAILRQLGGWPVVSDTWDQAQFTWLNSVYQFRKLGYSVDYFMDFSVATDAKNSTYRAINLDQASLGVSREYLVKGKEDPIVKAYRKYQVDVAVAFGANKERAEKELEESLDFEIKLANISLPSEKRRNITQLYNAIDVETLQRKYPSIPWLEYINNLLPAGIQVRNDEKVIIAVPSFIKELEKLLSVTPKRILANYVITRAVLSSVSYLTEDLRSNQLKFASALTGKMERESRWKECVDIVSGGVSLPVGSLYVRKYFKEEAKTAALEMVQDIRNEFVKILKTIDWMDEDTRIKGLEKADSMTVHIAYPDELLDDKKLDDFYNGLELDPQLYLASILNLTKFGTSYSFGRLRQKVNKTEWITHGRPAIVNAFYSSIENSIQFPAGILQGHFFGYDRPKYMNYGAIGFVIGHEITHGFDDQGRQFDKTGNLVDWWDPNTKQKYLQKAKCIIDQYGNYTEEMTGLKLNGINTQGENIADNGGIKEAYRAYLSWAERNGGEQRLPGLDFSPRQMFWISAAQSWCSKYRPEAMRQRITTGVHSPGRFRVLGPCSNVEEFSRDFDCPAGSRMNPAAKCSVW*

>Chv1VELVK21_0124071_translation Description: Membrane metallo-endopeptidase-like 1 [Zootermopsis nevadensis]

MSSCAAAGKGPAPGAEGTDARKSLVAGNANNGFLRVSSPGKDSEEQKVLLGQEVRVRENPLAAAATTVPRRRDAAGGRRCRRRRRRERVLVAVTAGLLLACCCFLLFGLLPREDCEAVIARKVCLTEECVRTASSLLVAMDRSADPCRDFFQFACGTWNKKHVIPEDRSSISTFEVLADQLQVILKGLLEEPKNEYDNAATVKAKRFYNSCMDIQQIRKNGDAPLREVLESLGGWPVTKSDWQPPSYSIETLLGRLRGEFNEGVLIEQWVGPDDKNSSVNILQLDQMQLALPGRDYYLKASSEGDLKAYHKYMTQVAVLLGANTTTAADELRHVIMFEKELANASLPEADRHDTSAIYRKLRLYELQKEVPQLNWLQYLKAFLDTDIDDGEPVVSYAMQYFVEMGRIIQRTDRRVIHNYVLWRLVMNIMPHMIDEYQQKSVDFKKILMGILSERNRWSQCVEWTNKKLGMAVGALFIRDNFNQESKETALEMIHTIRHAFNELLVENHWMDDDTRAVAKEKADAMNERIGYPELLTDAEELSKEYVMLNITEDQFLVNIL

NVLRYDAYHNLQKLRQPVNKDKWSTEPAVVNAFYNPNKNDIVFPAGILQPLFYSQHFPKSLNYGGIGVVIGHEITHGFDDKGRQFDKDGNMMQWWNNATVRAFRERAQCIIEQYSKYKLDEVGLFVNGRMTQGENIADNGGLKQSFRAYRKWVAEHGEEPGLPGMDLTHDQLFFLNYAQIWCGSMRPEDALTKIRSSVHSPGPIRVLGPLSNSHDFAAAYHCPLGSPMNPEDKCTVW*

**Gamma-glutamyltranspeptidase:**

>Chv1VELVK21_0017697_translation Description: PREDICTED: gamma-glutamyltranspeptidase 1-like isoform X3 [Tribolium castaneum]

MAQGWENVSLLQEFAAAGEDVRELFHEEYDMREWASKAAPAGRRGAALNMSFESEDGDSDSDERGYVKVPQKATASRDDGSCSDDDVEERWKPQTQRQRPCCRPKLQKRFVLLIGVVLLVCAAVGLVQLSYRLSAPSRPSLVPPDPEVPLPPSPSVEHRFRTAAVCTDGAPCSAVGKEILEKNGSVIDAAIAAMFCNGIVNSQSMGLGGGFLMTLYWRDRQTAETLNARESAPAAATADMFHGKPGLSQKGPLAIGVPGELKGYWEAHKKYGKLEWSALIEPSIRICEEGYNMTKHVQDSLDIRSSSVYDSPVLRATFVDPVTGKLKRQGALIRLGKMCSTLRTIAQRGGDDLYTGSLAKMLAEDIANMGGIITEEDLHNYKVQWQDPVSVALRDGVKVFSMPPPGSGVLLAFILNILDGYNFTRDSIGSAAATATTYHRIIEAYKYAYARRTELGDPQYVNVVELIRNLTSPEYASLIRKKIHDNATMNDASKYGAVFYNQDDHGTAHISIIAPNGDAVSVTSTVNIYFGAGVVSERTGIILNSGMDDFSVTGYNNYFGLPYSPANAIQPGKRAMSSMSPSILVDKSGDVRLVIGASGGTKITTATAYVILRYIWFQNTLKEAVDASRIHHQLFPMEVQYEYGVLDEVVKSLAKIGHNVTRYRDRGSIICAIAKEGDSIIANADYRKGGDVFGM*

>Chv1VELVK85_0006040_translation Description: Gamma-glutamyltranspeptidase 1 [Zootermopsis nevadensis]

MFLSLCRQKRGVLLLALGVLALSVTPVRPVDPTEPLPPSASPQGVYTRAAVATNGRPCADIARDILLKNGSAMEAAVAALFCEGVACPQSMGLGGGFLMTAYKRDTSHAYALDAREVAPAKATKDMYQKDSTLSQYGGLSVAVPGELKGYQALFDHKDFHSGNVEWSELVKPTIKLCEEGVEVTPYLAGILATREDVIKQSPTLSEILINNATGSVWRAGDKIKRPQLAQTLRVISKDGAAALYNGSLTDGFVKDIQSLGGIITEQDMASYSVRWEEPVTTTLDDGNLTLYSMPLSGSGHLVAFMLNMLDGFLPAERDTTAYHRLVESMKYAYGRRTQLGDVAFVHSIDDLLKNLTSKQYAEEVRGQVNDTMTWSDPAHYGAVLAQADDHGTAHISVLAPNGDAVSVTSTINHIFGSRVRSLSTGIILNNEMDDFSAPNMKNAYGLLPFPNNYIEPGKRPLSSMAPTIFVDRGGDVRLIVGAAGGSKITTATTEVSVNNLWWNMTLKEAVDASRLHHQLIPMELNYEKGFPKQYVMGLQQLGHQMRCLKTAGSTVTAISRQDGKVYANADYRRNGSIAGF*

>Chv1VELVK75_0011772_translation Description: Gamma-glutamyltranspeptidase 1 [Zootermopsis nevadensis]

MFLSLCRQKRDVLLLALGVLAVCTVALVVGLHFGQNPARSVTPVRLGDPIEPLPLSASPPGVYMRAAVATYSQPCADIARDILLKNGSAMEAAVAALFCEGVSSPQSMGLGGGFLMTAYKRDTGRAYALDAREVAPANATKDMYHGDSLLSQYGGLSVAVPGELKGYQALFDHKDFHSGNVEWSELVKPTIKLCEEGVEVSPYLADTLATREDVIKQSPTLIEILINNATGSVWRAGDKMKRPQLAQTLRVISKDGAAALYNGSLTDGFVKDIQSLGGIITEQDMASYSVRWEEPVTTTLDDGNLTLYSMPLSGSGHLVAFMLNMLDGFLPAERDTTAYHRLVESMKYAYGKRSQLGDVAFVHSIDDLLKNLTSKQYAEEVRRQVNDTMTWSDPAHYGAVLAQADDHGTGHISVLAPNGDAVSITSTINLIFGSRVRSLSTGIILNDEMDDFSAPNITNAYGLPPFPNNYIEPGKRPLSSMAPTIFVDRGGDVRLVVGAAGGSKITTSTTEVSVNNLWWNMTVKEAVDSSRLHHQLIPMELNYENGFPEQYVMGLQQLGHQMTRLETAGSAVTAISRQDGKIYSNADYRSNGSTAGF*

>Chv1VELVK25_0015659_translation Description: Glutamyl aminopeptidase [Zootermopsis nevadensis]

MSLKILPLFFCIHQEFPTSRCVRLFLRRFLVVFKLCAFYTYSSKRYQMLGDEAPSPTKAGWEADFRLPADVAPLRYHLLLHPDLEAGTFSGSVDVAVEVKAPRTWLAVNCKNLSVSGTRLLLVSGEEVPLASSFEYAPHEFWVAQTAAAIAPGEYVLRMDFAGQLTGKIIGFYRSVYTDPRAKGKRLIATSKFQPTYARQAFPCFDEPCFKATFNVRLVRPSDGYSALSNMDQTGEVADSPRPGLTTVQFRTSVPMVTYLVCFIVSDFQKLQPVQISQGFPVSVYSTPAQVNKTMFALGLGSKVTEYYIKYFGIPYPLPKLDLIAIPDFVSGAMEHWGLVTFRETVLLYDDTISSTTNKQRVALVVAHELAHMWFGNLMTLNWWSDLWLNEGFATYMEYKGVNSVHPEWHVMDQFLVEELHPVLSLDAVLSSHPIVQSVQHPDEITELFDTISYNKGASVIRMLEDFVGEERFQRGLSTFLKKFSFANAQTQDMWDAIQEVFSEANVTHVMDTWTRQMGFPVVTVKRTASGQLQLSQQRFLADPDANASAQTSPYNYRWDIPISYVTAESSEVNRTWFLSDQENVTITVPQSVAWVKLNHHQQGYYRVNYEPSLWTVFNSALSNNISILDAADRANLLDDAFKLANSGLLDYQMALDLSGYLQQETEYVPWAVASSNMAFLRSMLSSTTTYPKLRKYVRHLIQNIYNEVGWESSPNDSHLKKLLRVKVLSLACAYGLPECLQEVSRRFTAWVSDPGHVERPPPDLRNIIYQYGMYATSSEVIWDKLWGIFLDERDAQERLKLMYGLASVPEPWLLQRFIEYGKNESYVRSQDFFTLLHFISSNPVGNPIVWEFIRSEWQYLVSRFTLNDRYLGRMTSQVCGGFASHFKLQEMEAFFAKYPDAGAGATARRQALEKVSNNIKWHQRHRAVVDEWLTVAVQGLV*

>Chv1VELVK21_0024934_translation Description: Gamma-glutamyltranspeptidase 1 [Zootermopsis nevadensis]

MKYAYGKRSQLGDVAFVHSIDDLLKNLTSKQYAEEVRRQVNDTMTWSDPAHYGAVLAQADDHGTAHISVLAPNGDAVSVTSTINLIFGSRVRSLSTGIILNNEMDDFSAPNMKNAYGLLPFPNNYIEPGKRPLSSMAPTIFVDRGGDVRLIVGAAGGSKITTATTEVSVNNLWWNMTVKEAVDASRLHHQLIPMELNYEKGFPKQYVMGLQQLGHQMRCLKTAGSTVTAISRQDGKVYANADYRRNGSIAGF*

>Chv1VELVK21_0024932_translation Description: Gamma-glutamyltranspeptidase 1 [Zootermopsis nevadensis]

MVLMYLLKNLTSKQYAEEVRRQVNDTMTWSDPAHYGAVLAQADDHGTAHISVLAPNGDAVSVTSTINLIFGSRVRSLSTGIILNNEMDDFSAPNMKNAYGLLPFPNNYIEPGKRPLSSMAPTIFVDRGGDVRLIVGAAGGSKITTATTEVSVNNLWWNMTVKEAVDASRLHHQLIPMELNYEKGFPKQYVMGLQQLGHQMRCLKTAGSTVTAISRQDGKVYANADYRRNGSIAGF*

**Endoprotease FURIN:**

>Chv1VELVK45_0035041_translation Description: PREDICTED: furin-like protease 2 isoform X2 [Microplitis demolitor]

MMSAWLVVVALLLGRSLAGTSPTDPSKVKEVLSSETSTESSDPVQHFKNVPTTPEMSDPLFKDQWYLNGGAKGGLDMNVVPAWQKGYTGKGVVVSILDDGIQTNHPDLALNYDRLASTDINDNDDDPTPRDNGDNKHGTRCAGEVAGVAYNSICGIGVAYNASIGGVRMLDGTVNDAVEARALGLNPDHIDIYSASWGPEDDGKTVDGPGPLARRAFIYGVTKGRKGKGSIFVWASGNGGNHMDSCNCDGYTNSIFTLSISSVTQTGSKPWYLEECSSTLATTYSSGTPGRDRSVATVDMDLKLRPERLCTAEHTGTSASAPLAAGIVALALEANPRLSWRDVQYLVVLTSRSEPLRAEPGWAVNGVHRKVSHKFGYGLMDAAAMVSLAEKWTSVPPQHICKSQEVNKDMSISAAAGSVLTVQMDASGCAGRANEVSHLEHVQCKVSLRFLPRGNLRVLLTSPAGTTSVLLSERPRDLASSSFDEWLFLSVHHWGERPGGRWTLQVANSGEKRALFLCRRAEEVATHILRDSCEPRETESQREHGLLEVERARQPSHERSERERLAADQEEPELQEDAAARRQEQRTAAGQQLPPGVRPPGLLRSGAVPVPRLPALQTGQHVRAEVPPAQLP*

>Chv1VELVK21_0083474_translation Description: Endoprotease FURIN, putative [Pediculus humanus corporis]

MRGGSGRRRLQQHLRHRGGLQRQHWRGAHAGRHCERRSGGEGAGFESRPHRHLQASWGPEDDGKTVDGPGPLARRAFIYGVTKGRKGKGSIFVWASGNGGNHMDSCNCDGYTNSIFTLSISSVTQTGSKPWYLEECSSTLATTYSSGTPGRDRSVATVDMDLKLRPERLCTAEHTGTSASAPLAAGIVALALEANPRLSWRDVQYLVVLTSRSEPLRAEPGWAVNGVHRKVSHKFGYGLMDAAAMVSLAEKWTSVPPQHICKSQEVNKDMSISAAAGSVLTVQMDASGCAGRANEVSHLEHVQCKVSLRFLPRGNLRVLLTSPAGTTSVLLSERPRDLASSSFDEWLFLSVHHWGERPGGRWTLQVANSGEKRARDNGALKKWQLIFYGTAVNPVRLKANESMDFWKSNELANPLTNEVNVSDSLPIKKSPSYKKTLQLVDRNSVQLLANSCHPECDHQGCYGREPFQCLGCLHYKLDNTCVRRCPPRSFPNQEGRCWPCHESCETCAGPGQDSCVSCAPAHLLLADLATCLQQCPEGYYESYEQDSCIPCEPNCASCENRQCTRCDHHLFLYNNKCYATCPQFTYETGDHRCADCHASCQTCNGSGETQCISCRAGLFALDGACLAACPVRHFGHAKQRECVACPKGCSACDPVACTSCSPGWVLARTGWCIPLHSNSCSAGEFWDGYGCQRCDASCETCEGPASDQCLSCGYPRLQQGSQCVAACTGGYYAGRRGCLPCPHTCAACAARNNCTRCRLGLQLQTGECRATCAAGYYSDRGLCAKCHLSCMTCNGPRRDQCVLCPDDWELVEGECQPECPGGYFKLQSGCQKCHHYCRTCYGTGPLNCTSCGDKYVLDGGTCVPCLSGQYYAAVSQACAPCHDSCRSCSGPGPFSCSSCEPPLRLDGAAGQCVLCCSGDDAAPYCCSCDPATGDCEKPSHAQKRHIAQQVTLIFGKEESSDNRSTEYSVIVLKVSIAAIVFIGLVFGLVRVKHWSANPGWDKDDVCENLFTCQLRLTLEKMVSAKSTSR*

**Carboxypeptidase:**

>Chv1VELVK21_0032458_translation Description: Carboxypeptidase M [Zootermopsis nevadensis]

MKTTTWCASSLVVVFLYLYVMAVQYVRAAEAEDGGRVLFQGPEEGQQRVLTRPGVAPPAAADREEDEPAESSASLRAVNSNYQPGYSVDSAYGQYYGDSAGSATSADSSYNLEFKYHNYEQLTKFLRTTSSRFPNLTALYSIGKSVQGRDLWVMVVSASPYEHMIGKPDVKYVANMHGNEAVGRELMLHLVHYLVTSYNTDPYIKWLLDNTRIHILPSMNPDGFEVARESQCDGGQGRYNARGFDLNRNFPDYFKQNNKRGQPETDAVKEWISKIQFVLSGGLHGGALVASYPFDNTPNSSPVFHSYSAAPSLTPDDDVFKHLSLTYSSNHPVMRRGVACKAGTPAFNQGITNGAAWYPLIGGMQDFNYVWYGCMEVTLELSCCKYPPATELPKFWEENRTPLVKFLAEAHRGAQGFVMDENGNPVEKASLKVKGRDVGFQSTKYGEFWRILLPGVYKLEVYADGYVPREIDFMVVDQHPTLLNVTLHPTKLKNVEAADRQDNFYRPQPHHVYYHHAQGQHTKPAESGGIFSSISSGFNNLVSNIFG*

>Chv1VELVK65_0051556_translation Description: carboxypeptidase [Eupolyphaga sinensis]

MVTDGVAVVCLLLTAACCSGWGGAGDSQSAHNVSYRRPHFDKVAKRSHGNDYTRYSVFRLRPTEEQRQLVRGFYNKEGFDVWARGARNYTYVDIMVYPAKKRSFEDFLVRNNIAHTILIENVQETLKRETANLTRPCGGLFCQYNRYDTITRFMRETERRRPRQVRVESLGLTHEGRDILVMQISGDGGAAGRPVVLLEAGVHAREWIAPAAALRVMQGLAEDPRQADLLRHLDWHVVPVLNPDGYEYSYTTSRFWRKNRGKNKGSQCRGNDINRNFDFHWNEVGTSPDPCSDGYPGPRPFSSLESIALKNYALKHKHRIKLYLSLHSHGSYILYPWGYATELPSDWSLLHSVAVKADEAQVKAGGLPYVIGSSAKTLYPAAGCTDDWMKAVCGVNLSYTVELPGGGKRGFDLPPEYIEDTVNNFFHAVRAFGKELERLLLSSIL*

>Chv1VELVK65_0051554_translation Description: carboxypeptidase [Coptotermes formosanus]

MVTDGVAVVCLLLTAACCSGWGGAGDSQSAHNVSYRRPHFDKVAKRSHGNDYTRYSVFRLRPTEEQRQLVRGFYNKEGFDVWARGARNYTYVDIMVYPAKKRSFEDFLVRNNIAHTILIENVQETLKRETANLTRPCGGLFCQYNRYDTSRFWRKNRGKNKGSQCRGNDINRNFDFHWNEVGTSPDPCSDGYPGPRPFSSLESIALKNYALKHKHRIKLYLSLHSHGSYILYPWGYATELPSDWSLLHSVAVKADEAQVKAGGLPYVIGSSAKTLYPAAGCTDDWMKAVCGVNLSYTVELPGGGKRGFDLPPEYIEDTVNNFFHAVRAFGKELERLLLSSIL*

>Chv1VELVK55_0060004_translation Description: carboxypeptidase [Eupolyphaga sinensis]

MVTDGVAVVCLLLTAACCSGWGGAGDSQSAHNVSYRRPHFDKVAKRSHGNDYTRYSVFRLRPTEEQRQLVRGFYNKEGFDVWARGARNYTYVDIMVYPAKKRSFEDFLVRNNIAHTILIENVQETLKRETANLTRPCGGLFCQYNRYDTITRFMRETERRRPRQVRVESLGLTHEGRDILVMQISGDGGAAGRPVVLLEAGVHAREWIAPAAALRVMQGLAEDPRQADLLRHLDWHVVPVLNPDGYEYSYTTSRFWRKNRGKNKGSQCRGNDINRNFDFHWNEVGTSPDPCSDGYPGPRPFSSLESIALKNYALKHKHRIKLYLSLHSHGSVSIGLHCV*

**Signal peptidase complex subunit 2:**

>Chv1VELVK65_0011192_translation Description: putative sig-l peptidase complex subunit 2 [Zootermopsis nevadensis]

MSSKGGKDQDKPVKVNKWDGSAVKNALDDAVKEVLTKKFNYLESFALMDGRLAMCGIAVGVAMFALLWDYLYPFPQSRPILIFCVSAYFVMMGILTLYTTYKEKGIFVIAVQKDQAGFVPDNVWEASSFLKKFDDKYNLILSYKNGKTGSRYEATIVKSVANFFDENGTLVYELLEPEVSKLHNSLLAERKEK*
