# Supplementary material for: De Novo Transcriptome Analysis of the Common New Zealand Stick Insect Clitarchus hookeri (Phasmatodea) Reveals Genes Involved in Olfaction, Digestion and Sexual Reproduction
Source: PLoS One. 2016 Jun 23;11(6):e0157783. doi: 10.1371/journal.pone.0157783 (PMC4919086; doi:10.1371/journal.pone.0157783)
Supplement: S3 Text — (DOCX) [file pone.0157783.s004.docx]

| **ID (given in the study)** | **Nucleotide Accession No.** |
| --- | --- |
| *OBP15* | KDR21034.1 |
| *OBP1* | KDR09909.1 |
| *OBP2* | KDR09911.1 |
| *OBP3* | KDR09910.1 |
| *OBP14* | KDR13203.1 |
| *OBP5* | KDR13658.1 |
| *OBP6* | KDR13657.1 |
| *OBP7* | KDR13656.1 |
| *OBP8* | KDR13655.1 |

**Table 1** Accession IDs of the *Zootermopsis nevadensis* sequences downloaded from GenBank for constructing OBP protein phylogeny

**Table 2** Accession IDs of the sequences downloaded from GenBank for constructing ORCO protein phylogeny and performing branch selection test on coding sequences

| **No.** | **Species** | **GenBank Accession No. (Protein/mRNA)** |
| --- | --- | --- |
| 1 | *Musca domestica* | AFH96944 / JQ365179 |
| 2 | *Stomoxys calcitrans* | AGC69747 / JX996042 |
| 3 | *Chrysomya rufifacies* | AFH96943 / JQ365176 |
| 4 | *Ceratitis capitata* | AAX14775 / AY843206 |
| 5 | *Bactrocera dorsalis* | ACC86853 / EU621792 |
| 6 | *Drosophila melanogaster* | AAT71306 / AY567998 |
| 7 | *Aedes aegypti* | Q178U6 / XM_001651376 |
| 8 | *Anopheles gambiae* | AAX14774 / AY843205 |
| 9 | *Heliothis viriplaca* | AFI25169 / JQ394904 |
| 10 | *Helicoverpa armigera* | ADQ13177 / HQ186284 |
| 11 | *Agrotis segetum* | AGS41440 / KC526964 |
| 12 | *Lymantria dispar asiatica* | AHA50097 / KF482410 |
| 13 | *Conogethes punctiferalis* | AGF29886 / JX101681 |
| 14 | *Planotortrix excessana* | AJE25910 / KM678337 |
| 15 | *Plutella xylostella* | ACX54944 / GQ923610 |
| 16 | *Anomala corpulenta* | AKC58535 / KM251654 |
| 17 | *Holotrichia parallela* | AEG88961 / JF826514 |
| 18 | *Ambrostoma quadriimpressum* | AJF94638 / KM893996 |
| 19 | *Tenebrio molitor* | AJO62219 / KP296755 |
| 20 | *Macrocentrus cingulum* | AGI62937 / JX472452 |
| 21 | *Cephus cinctus* | AGS43074 / KC778527 |
| 22 | *Chouioia cunea* | AIY24336 / KJ847283 |
| 23 | *Locusta migratoria* | AEX28370 / JN989549 |
| 24 | *Schistocerca gregaria* | AEX28371 / JN989550 |
| 25 | *Adelphocoris fasciaticollis* | AHC72294 / KC881259 |
| 26 | *Lygus lineolaris* | AFX73448 / JQ639214 |
| 27 | *Apolygus lucorum* | AHC72290 / KC881255 |
| 28 | *Cimex lectularius* | AIW42735 / KM275232 |

**Table 3** Accession IDs of nucleotide coding sequences downloaded from GenBank for constructing GH 9 phylogeny

| **No.** | **Species** | **Nucleotide Accession No.** |
| --- | --- | --- |
| 1 | *Tribolium castaneum* | XM_001810641.1 |
| 2 | *Sinocapritermes mushae* | AB118806.1, AB118805.1, AB118804.1 |
| 3 | *Salganea esakii* | AB438948.1, AB438947.1, AB438946.1 |
| 4 | *Reticulitermes speratus* | AB019095.1, AB008778.2, AY572862.2 |
| 5 | *Polyphaga aegyptiaca* | AF220583.1, AF220585.1, AF220584.1 |
| 6 | *Periplaneta americana* | AF220587.1, AF220586.1 |
| 7 | *Pediculus humanus corporis* | XM002426420.1 |
| 8 | *Panesthia cribrata* | AF220597.1, AF220596.1 |
| 9 | *Panesthia angustipennis* | AB438952.1, AB438951.1, AB438950.1 |
| 10 | *Odontotermes formosanus* | AB118802.1, AB118801.1, AB118800.1, GU326330.1 |
| 11 | *Neotermes koshunensis* | AB118799.1, AB118798.1, AB118797.1, AF220591.1 |
| 12 | *Nasutitermes walkeri* | AB013273.1 |
| 13 | *Nasutitermes takasagoensis* | AB118803.1, AB013272.2 |
| 14 | *Nasonia vitripennis* | XM001606404.2 |
| 15 | *Mastotermes darwinensis* | AJ511343.1, AJ511342.1, AJ511341.1, AJ511340.1, AJ511339.1 |
| 16 | *Hodotermopsis sjoestedti* | AB118796.1, AB118795.1, AB118794.1, AB118662.1 |
| 17 | *Hodotermopsis japonica* | AF220592.1 |
| 18 | *Cryptocercus clevelandi* | AF220588.1, AF220589.1, AF220590.1 |
| 19 | *Coptotermes formosanus* | AB058671.1, GU017483.1, AB058670.1, AB058669.1 |
| 20 | *Coptotermes acinaciformis* | AF336120.1 |
| 21 | *Blattella germanica* | AF220595.1 |
| 22 | *Apis mellifera* | XM396791.4 |
| 23 | *Acyrthosiphon pisum* | XM001944739.2 |
